# Use of multimodal dataset in AI for detecting glaucoma based on fundus photographs assessed with OCT: focus group study on high prevalence of myopia

**DOI:** 10.1186/s12880-022-00933-z

**Published:** 2022-11-24

**Authors:** Wee Shin Lim, Heng-Yen Ho, Heng-Chen Ho, Yan-Wu Chen, Chih-Kuo Lee, Pao-Ju Chen, Feipei Lai, Jyh-Shing Roger Jang, Mei-Lan Ko

**Affiliations:** 1 Department of Computer Science and Information Engineering, National Taiwan University, Taipei City 10617, Taiwan, ROC; 2 School of Medicine, National Taiwan University, Taipei City 10617, Taiwan, ROC; 3 Department of Applied Mathematics, National Sun Yat-Sen University, Kaohsiung City 804201, Taiwan, ROC; 4 Department of Internal Medicine, National Taiwan University Hospital Hsin-Chu Branch, Hsinchu City 300, Taiwan, ROC; 5 Department of Electrical Engineering, National Taiwan University, Taipei City 10617, Taiwan, ROC; 6 Department of Ophthalmology, National Taiwan University Hospital Hsin-Chu Branch, No. 25, Lane 442, Sec.1, Jingguo Rd., Hsinchu City 300, Taiwan, ROC; 7 Department of Biomedical Engineering and Environmental Sciences, National Tsing Hua University, Taipei City 10617, Taiwan, ROC

**Keywords:** Deep learning, Glaucoma, Multimodal learning model, Ophthalmology

## Abstract

**Background:**

Glaucoma is one of the major causes of blindness; it is estimated that over 110 million people will be affected by glaucoma worldwide by 2040. Research on glaucoma detection using deep learning technology has been increasing, but the diagnosis of glaucoma in a large population with high incidence of myopia remains a challenge. This study aimed to provide a decision support system for the automatic detection of glaucoma using fundus images, which can be applied for general screening, especially in areas of high incidence of myopia.

**Methods:**

A total of 1,155 fundus images were acquired from 667 individuals with a mean axial length of 25.60 ± 2.0 mm at the National Taiwan University Hospital, Hsinchu Br. These images were graded based on the findings of complete ophthalmology examinations, visual field test, and optical coherence tomography into three groups: normal (N, n = 596), pre-perimetric glaucoma (PPG, n = 66), and glaucoma (G, n = 493), and divided into a training-validation (N: 476, PPG: 55, G: 373) and test (N: 120, PPG: 11, G: 120) sets. A multimodal model with the Xception model as image feature extraction and machine learning algorithms [random forest (RF), support vector machine (SVM), dense neural network (DNN), and others] was applied.

**Results:**

The Xception model classified the N, PPG, and G groups with 93.9% of the micro-average area under the receiver operating characteristic curve (AUROC) with tenfold cross-validation. Although normal and glaucoma sensitivity can reach 93.51% and 86.13% respectively, the PPG sensitivity was only 30.27%. The AUROC increased to 96.4% in the N + PPG and G groups. The multimodal model with the N + PPG and G groups showed that the AUROCs of RF, SVM, and DNN were 99.56%, 99.59%, and 99.10%, respectively; The N and PPG + G groups had less than 1% difference. The test set showed an overall 3%–5% less AUROC than the validation results.

**Conclusion:**

The multimodal model had good AUROC while detecting glaucoma in a population with high incidence of myopia. The model shows the potential for general automatic screening and telemedicine, especially in Asia.

*Trial registration*: The study was approved by the Institutional Review Board of the National Taiwan University Hospital, Hsinchu Branch (no. NTUHHCB 108-025-E).

**Supplementary Information:**

The online version contains supplementary material available at 10.1186/s12880-022-00933-z.

## Introduction

### Background

Glaucoma, a neurodegenerative disease, is a significant cause of blindness worldwide. Due to ambiguous symptoms, early diagnosis is difficult. When signs develop, the visual field (VF) is usually lost. Chua et al. estimated that approximately 50% of patients with glaucoma were undiagnosed [1]. Accurate glaucoma detection is based on a combination of clinical examinations and various tests for structural and functional optic nerve head damage, including fundus photography, visual field test, optical coherence tomography (OCT) [2]. However, most of these devices are only available in regional hospitals or medical centers. Fundus examination is a basic tool commonly used for the diagnosis of glaucoma. In addition, some prospective research about taking fundus photos with only a smartphone has appeared recently [3], which is prone to increase the convenience and prevalence of fundus photos in the future. However, examining glaucoma through fundus photography requires considerable clinical experience, and the conclusions often differ from those of experts [4, 5]. Therefore, artificial intelligence (AI) methods might have great potential to address this problem because of the recent well-established convolutional neural networks to process big data with increased processing speed and accuracy.

Over the past few years, AI has been widely applied to different aspects of ophthalmology [6–10], in which glaucoma is one of the most popular. Researchers have developed several algorithms to differentiate glaucomatous eyes from normal eyes using fundus photography to detect glaucoma. The areas under the AI receiver operating characteristic curves (AUROCs) in the studies by Ting DSW et al., Li Z et al., and Christopher M et al. were 0.942, 0.91, and 0.986, respectively [11–13]. However, these studies did not focus on the increasing prevalence of myopia.

Myopia may cause morphological changes in the retina and optic disc. For instance, retina tessellation, staphyloma, optic disc tilting, and peripheral papillary atrophy [14, 15] may make the diagnosis of glaucoma through the fundus more difficult. Li et al. found that when AI differentiates glaucomatous images from non-glaucomatous ones, pathologies or high myopia are the most common causes of false-negative results [12]. Myopia also plays an important role in the false-positive results. The condition is prevalent worldwide. According to a previous study, the global prevalence of myopia will reach 49.8% in 2050, which is more than twice the prevalence reported in 2000 [16]. In Taiwan, from 2010 to 2011, the prevalence of myopia in men between 18 and 24 years of age was 86.1% [17]. Therefore, the high prevalence of myopia will become an obstacle for AI to diagnose glaucoma.

## Objective

This study aims to provide a tool that can help diagnose glaucoma by using color fundus images, which can be applied in areas with a low level of medical resources or areas where many people have myopia.

## Methods

This study was conducted at the National Taiwan University Hospital, Hsinchu Branch, Taiwan, in accordance with the Declaration of Helsinki (1964). The study was approved by the Institutional Review Board of the National Taiwan University Hospital, Hsinchu Branch (no. NTUHHCB 108-025-E). Informed consent was obtained from all participants.

In order to make the proposed methodology clearer we listed the main checkpoints in our methods as follows.

1. Collecting data and precise glaucoma grading with optical examinations, fundus images, visual field, and OCT data
2. Selecting the Xception transfer learning model as the fundus image classifier.
3. Training a fundus image regression model to predict OCT-obtained RNFLT and C/D vertical ratios
4. Training a multimodal model using the predicted RNFLT, C/D vertical ratios (both from the regression model), color fundus images, and optical examination numerical results
5. Performing error analysis to identify false predictions and improve model training

### Subjects and data collection

All data were collected when the participants visited the hospital's general or ophthalmologic clinic from June 2019 to September 2020. After providing informed consent, demographic data was collected (for example, age and sex) and the participants underwent a set of ophthalmologic examinations, including a visual acuity test using a Snellen chart, measurement of intraocular pressure (IOP) using a non-contact tonometer NT-530P (Nidek Co., Gamagori, Japan), measurement of refractive error using an ARK-510A (Nidek Co., Gamagori, Japan), measurement of axial length using an AL-SCAN (Nidek Co., Gamagori, Japan), slit-lamp examination, gonioscopy, fundoscopy, and a visual field test using a Humphrey Field Analyzer-840 (HFA-840; Carl Zeiss Meditec, Inc. Dublin, CA, USA). Both 45° optic disc-centered and macular-centered color fundus images with a resolution of 1620 × 1440 were captured using a Zeiss VISUCAM 524 (HFA-840; Carl Zeiss Meditec, Inc. Dublin, CA, USA) in the color and green modes. Both eyes of each participant were evaluated and imaged.

All participants underwent OCTA (Angiovue, Optovue Inc., Fremont, CA, USA) using the split-spectrum amplitude-decorrelation angiography algorithm. All images contained retinal nerve fiber layer RNFL thickness, ganglion cell complex thickness, and optic nerve head analysis, including the cup/disc ratio, rim area, and disc area. The RNFL thickness measurement was centered on the optic disc in an annulus region, with the outer diameter as 4 mm and the inner diameter as 2 mm. The GCC scan, comprising retina nerve fiber, ganglion cell, and inner-plexiform layers, was centered 1 mm temporal to the fovea with a scan size of 7 mm × 7 mm area. All scans were reviewed manually to ensure correct disc/cup segmentation and quality. The RNFL thickness and ganglion cell complex thickness calculation were divided into superior and inferior parts, to find compatible glaucomatous defects in the visual field examination. Subjects with or without pseudophakia between 20 and 80 years of age were included. Any subjects with positive cataract would be excluded due to blurred color images. The exclusion criteria included: best-corrected visual acuity below 12/20; a history of ocular disease, such as vitreoretinal diseases, ocular trauma, uveitis, non-glaucomatous optic neuropathy, retinopathy, and any other ocular disease that may affect optic nerve or visual field result; systemic diseases, such as diabetic retinopathy, hypertensive retinopathy, stroke with visual field loss; OCTA scan signal strength index less than 40; visual field test: fixation loss > 15%, false positive rate > 20% or and false negative > 10%

A grading system was established using OCT numerical data extracted from OCTA and visual field examination. According to the ganglion cell complex and RNFL thickness, OCT numerical data showed normal, borderline, and abnormal ganglion cell complex values in the macular and optic nerve head fiber layers of the image. The borderline and normal categories were combined into the normal category to simplify the grading system. All participants were diagnosed with normal (N), pre-perimetric glaucoma (PPG), or glaucoma (G). Specifically, PPG is the case where either the ganglion cell complex, RNFL, or both regions were damaged, but the visual field was still normal. For the G group, the ganglion cell complex, RNFL, or both regions were damaged and showed compatible visual field changes. The mean defect of visual field more than -2 dB would be considered as abnormal visual field. Our study team have built up a normal ground of OCT and OCTA for normal subjects with different axial length [18]. All glaucoma patients were followed more than 1 years and their collected data was confirmed by an experienced glaucoma specialist. It is worth mentioning that all glaucoma patients had been followed up with for at least one year. The analysis collapsed the three groups into binary groups, N + PPG, G and N, PPG + G, to form non-glaucomatous and glaucomatous groups. Note that each image corresponds to a unique person. We sorted the collected data in chronological order, and then took the data from the last two weeks as the test set and the rest as the training set.

### Preprocessing of images and numerical data

The fundus images were automatically cropped by only keeping the pixels within the red circle, as shown in Additional file 1. The images were cropped using the OpenCV package [19]; details are provided in Additional file 1. Most of the numerical data from optic examination, OCTA, and patient demographics had missing ratios below 5%; all the missing values were filled by the MICE algorithm [20].

### Transfer learning image classification

This study applied transfer learning model with Xception as base model using ImageNet dataset training weights. The last output layer was replaced with a dense layer for classification. A self-attention model was applied using the method used by Guan et al. [21]. Self-attention methods can help our models identify key regions of fundus images associated with glaucoma. The key regions identified by self-attention methods are cropped, and both the cropped image and the whole image were sent to two Xception models initialized by pretrained weights. Each Xception model generates a feature map (or output vector) of 1 × 1024. These two feature maps are concatenated to form a vector of 1 × 2048, which is then sent to a fully connected neural networks of 5 hidden layers to generate an output for predicting glaucoma. Details of the data processing are shown as a block diagram under the self-attention section in Fig. 2. All the convolution layers and last classification layer were fine-tuned [22]. The Adam optimizer was used in the model with a learning rate of 0.0001 and a decay of 0.001 with categorical cross-entropy as the loss function. All of the models were trained for 80 epochs in batches of 30 images per step with ten-fold cross-validation for model evaluation. The validation accuracy was monitored during the model training process, and the model was saved with the best validation accuracy to predict the test data. First, a single transfer learning image classification model was trained with both the disc center and macular center images for selecting the best base models from Inception v3, Inception Resnet v2 and Xception [23, 24]. After the selection we trained both disc-center and macular-center transfer learning images classification models separately and used them in the multimodal model. All models were trained on a computer with Ubuntu (16.04.6), Intel(R) i7-7740X CPU, two GeForce GTX 1080 Ti 11 GB GPU, and 62GiB system memory.

### Regression model for predicting the OCTA-acquired C/D v ratio and average RNFL thickness

To include essential features such as the cup/disc vertical ratio (C/D v ratio) and average RNFL thickness, we used a regression model that included color fundus images as input to predict the OCTA-acquired C/D v ratio and average RNFL thickness with a method similar to that used by Medeiros et al. [25]. The color fundus images were magnified onefold to the optic disc area and manually cropped to increase the model accuracy. The regression model was trained using the transfer learning method with the Xception network. The last output layer was changed to a dense layer with a linear activation function and mean-squared error loss function for value prediction.

### Multimodal model

Patients' demographic data, OCT numerical data extracted from OCTA, and color fundus images were collected. The data collection consisted of numerical data and two images: disc-centered and macular-centered color fundi. Numerical data, alongside disc-centered and macular-centered color fundus images, were included in the training input. The numerical data included age, sex, axial length, visual acuity, heart rate, and blood pressure. Glaucoma specialists selected these features, considering that most of the subjects in this study had high myopia. The regression model predicted the C/D v ratio and average RNFL thickness as the input of the multimodal model. After training the models with disc-centered color fundus and macular-centered color fundus, the feature maps extracted by the second last layer were concatenated with the selected numerical features. Random forest (RF), support vector machine (SVM), Ada-boost (Ada), decision tree (with either the CART or C4.5 algorithm), logistic regression (LogReg), Naïve Bayes (NB), k-nearest neighbors (KNN), and a dense neural network (DNN) were trained with the newly concatenated inputs for the final prediction. Finally, a webpage was built with the multimodal model we proposed for general screening of glaucoma and telemedicine application. An illustration of the process described above was shown in Additional file 2.

### Statistics

All statistics were computed using Python packages, Scikit-learn and NumPy. True-positive rate (sensitivity)–false-positive rate (1—specificity) receiver operating characteristic (ROC) curves were plotted. Using the ROC curve, the tradeoffs between sensitivity and specificity, and the area under the ROC (AUROC) curve was used to determine the model's performance. Accuracy, F-measure, and confusion matrix were computed based on the ROC curve's optimal cutoff point using Youden's J statistic [26]. All the metric equations were listed in Additional file 3.

## Results

### Study design flow and proposed models

To design a decision-making model for precise glaucoma diagnosis, both disc-centered and macular-centered images should be completed. The dataset in Table 1 was acquired after excluding blurred, ambiguous images and images that only had one single-centered image. Our models predict the probabilities of normal, PPG, or glaucoma as outcomes. These possibilities were regrouped into either (normal + PPG) and glaucoma, or normal and (PPG + glaucoma). The models required the following predictors (features) to predict the outcome: disc-and macular-centered RGB color fundus images (RGB channels with normalized 0–1 values), C/D v ratio (%), RNFL thickness (μm), age, sex (0 = female, 1 = male), visual acuity (logMAR), axial length (mm), heart rate (bpm), systolic blood pressure (mmHg), and diastolic blood pressure (mmHg); the full features were listed in Table 1. The correlation matrix of the features was calculated and shown in the Additional file 4.

**Table 1:** Participants' demographic data

Features	Control eyes (n = 596)	PPG eyes (n = 66)	Glaucoma eyes (n = 493)	p-value
Age (years), mean ± SD	45.3 ± 14.2	45.7 ± 12.7	51.8 ± 13.6	< .001
Age (years), n (%)				
< 40	221 (37.1)	20 (30.3)	100 (20.3)	
40–60	295 (49.5)	39 (59.1)	258 (52.3)	
≥ 60	80 (13.4)	7 (10.6)	135 (27.4)	
Gender, n (%)				< .001
Male	210 (35.2)	43 (65.2)	323 (65.5)	
Female	386 (64.8)	23 (34.8)	169 (34.3)	
OD/OS, n (%)				.76
OD^a^	301 (50.5)	35 (53.0)	241 (48.9)	
OS^b^	295 (49.5)	31 (47.0)	252 (51.1)	
Visual acuity (logMAR), mean ± SD	0.9 ± 0.2	0.9 ± 0.2	0.8 ± 0.3	< .001
Axial length (mm), mean ± SD	25.19 ± 1.78	25.93 ± 1.84	25.67 ± 2.23	< .001
Axial length (mm), n (%)				
< 24	163 (27.3)	13 (19.7)	126 (25.6)	
24–25.9	231 (38.8)	15 (22.7)	148 (30.0)	
≥ 26	199 (33.4)	38 (57.6)	218 (44.2)	
IOP^c^ (mmHg), mean ± SD	14.7 ± 3.4	15.0 ± 3.9	14.4 ± 3.8	.30
CCT^d^ (μm), mean ± SD	543.6 ± 35.6	531.8 ± 43.4	534.9 ± 35.9	< .001
Visual field: mean defect (dB), mean ± SD	− 1.2 ± 1.8	− 1.8 ± 4.5	− 10.7 ± 9.6	< .001
Macular VD^e^ (%), mean ± SD				
Superior	50.1 ± 4.9	46.6 ± 6.5	42.2 ± 7.6	< .001
Center	18.5 ± 6.4	18.1 ± 6.1	15.7 ± 6.8	< .001
Inferior	49.5 ± 5.2	46.0 ± 5.9	39.5 ± 8.2	< .001
Disc VD (%), mean ± SD				
Superior	51.6 ± 4.9	47.4 ± 5.8	37.1 ± 11.0	< .001
Inferior	52.5 ± 5.3	47.6 ± 5.0	34.6 ± 10.5	< .001
RNFL^f^ thickness (μm), mean ± SD				
Superior	100.2 ± 9.9	86.8 ± 10.4	74.5 ± 15.5	< .001
Inferior	96.2 ± 9.2	82.0 ± 10.7	67.7 ± 14.6	< .001
Ganglion cell complex thickness (μm), mean ± SD				
Superior	95.5 ± 5.9	86.0 ± 7.8	75.4 ± 12.7	< .001
Inferior	95.0 ± 6.2	81.5 ± 8.0	69.2 ± 12.5	< .001
Cup/Disk Vertical Ratio (%), mean ± SD	52.0 ± 19.6	69.5 ± 16.0	83.1 ± 14.0	< .001
Rim Area (0.01 mm^2^), mean ± SD	130.2 ± 38.2	100.1 ± 50.2	66.8 ± 35.6	< .001
Disc Area (0.01 mm^2^), mean ± SD	206.0 ± 49.8	213.9 ± 49.8	214.5 ± 62.0	.03
SBP^g^ (mmHg), mean ± SD	124.1 ± 18.5	128.5 ± 16.0	128.2 ± 17.9	< .001
DBP^h^ (mmHg), mean ± SD	73.4 ± 12.5	78.2 ± 12.3	76.1 ± 12.0	< .001
MAP^i^ (mmHg), mean ± SD	90.3 ± 13.5	95.0 ± 12.6	93.5 ± 12.8	< .001
HR^j^ (/min), mean ± SD	79.1 ± 12.4	75.1 ± 10.5	75.1 ± 12.4	< .001

^a^OD: right eye

^b^OS: left eye

^c^IOP: intraocular pressure

^d^CCT: central corneal thickness

^e^VD: vascular density

^f^RNFL: retinal nerve fiber layer

^g^SBP: systolic blood pressure

^h^DBP: diastolic blood pressure

^i^MAP: mean arterial pressure

^j^HR: heart rate

**Fig. 1:**
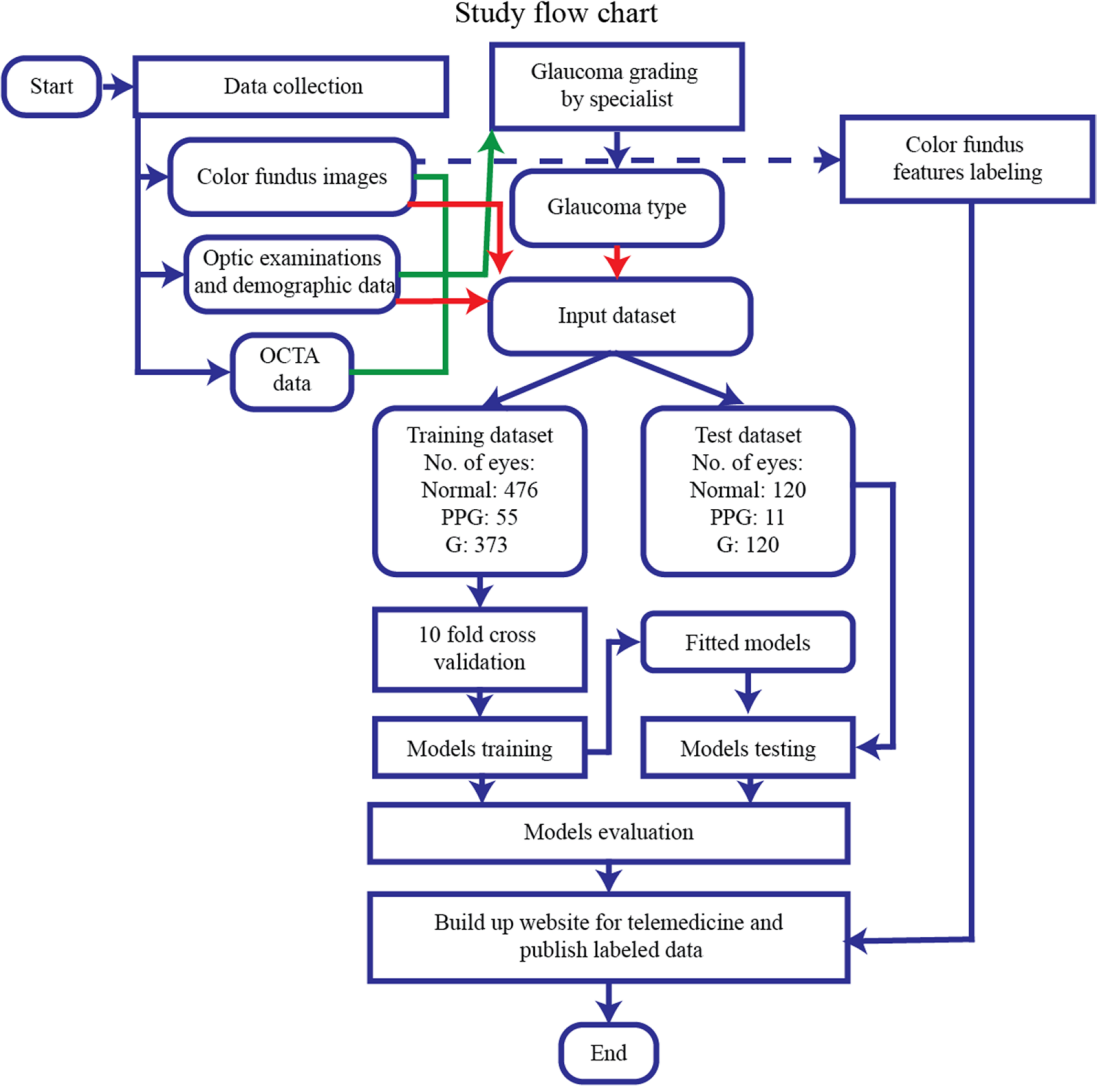
The flow chart of the study design. First, dataset collection was done at the National Taiwan University Hospital, Hsinchu Branch. After glaucoma specialists reviewed all the collected images as well as the participants’ demographic and OCT extracted numerical data, each participants’ eye was precisely graded as N, PPG, or G. OCT extracted numerical data were not included in the training process. Data from the last two weeks was kept as a test dataset, and the rest of the data were used for training the models with tenfold cross-validation. The model was built on a webpage for telemedicine and the labeled color fundus images will be published as open data. N, normal; OCTA, optical coherence tomography angiography; PPG, pre-perimetric glaucoma; G, glaucoma

**Fig. 2:**
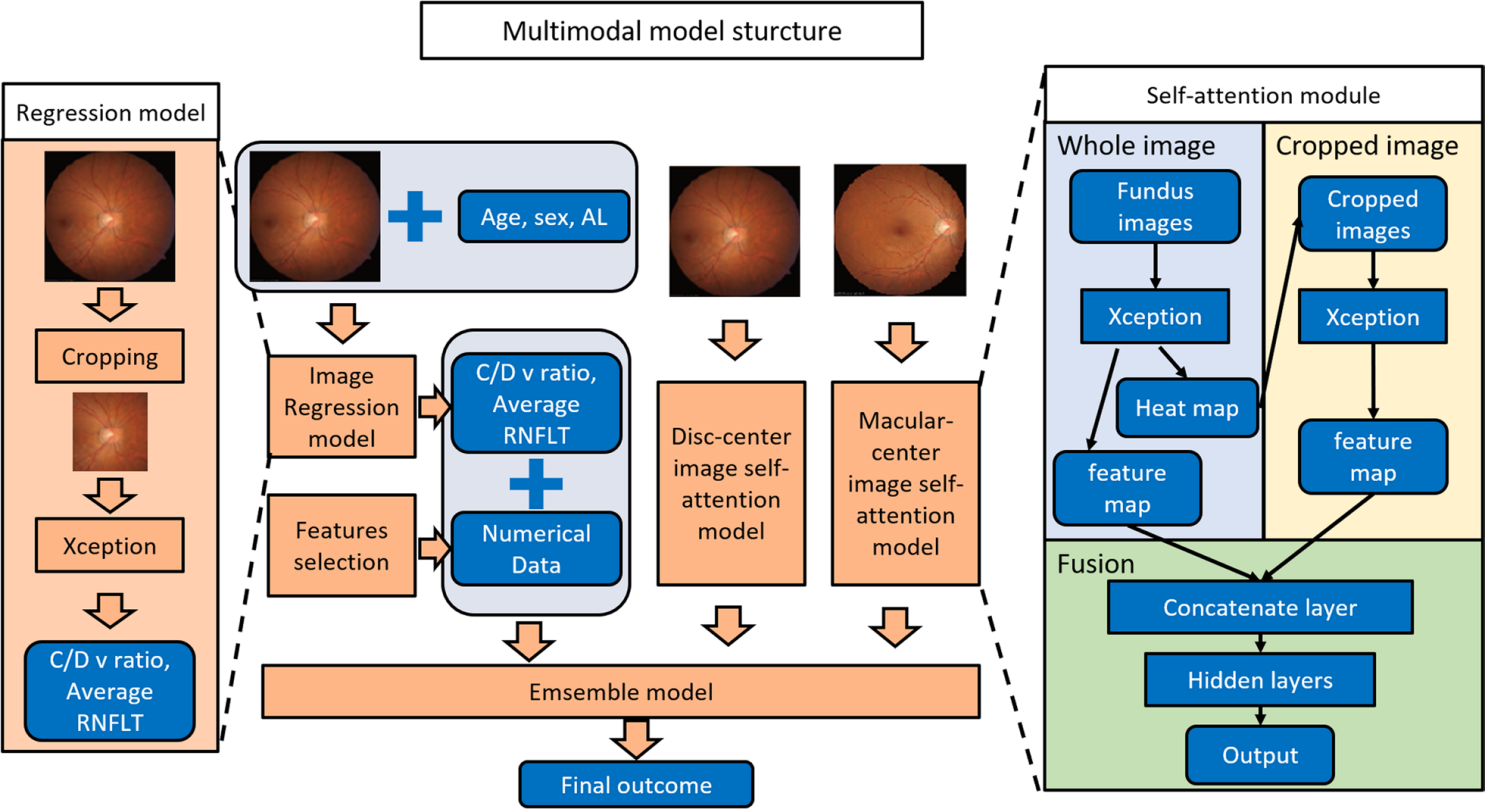
The layout of the proposed model. The multimodal model was combined with two image classifiers (disc-view color fundus, macular-view color fundus), a regression model (C/D V ratio, and average RNFLT), axial length, VA, and participants’ demographic numerical data. C/D V ratio, Cup/Disc vertical ratio; RNFLT, retinal nerve fiber layer thickness; VA, visual acuity

The subjects who participated in this study had an overall mean axial length of 25.19 ± 1.78 mm, 25.93 ± 1.84 mm, 25.67 ± 2.23 mm in N, PPG, and G groups, respectively, as shown in Table 1. Eye with a spherical equivalent ≤ -6.0 diopter (D) or an axial length (AL) $\ge$ 26 mm is defined as high myopia [27]. The number of participants with high myopia (axial length ≥ 26 mm) was 199 out of 596 (33%) in the N group, 38 out of 66 (57%) in the PPG group, and 218 out of 493 (44%) in the G group. After grading and preprocessing the data, the study design flow (Fig. 1) was followed to train the proposed model, as shown in Fig. 2.

### Transfer learning image classification

**Fig. 3:**
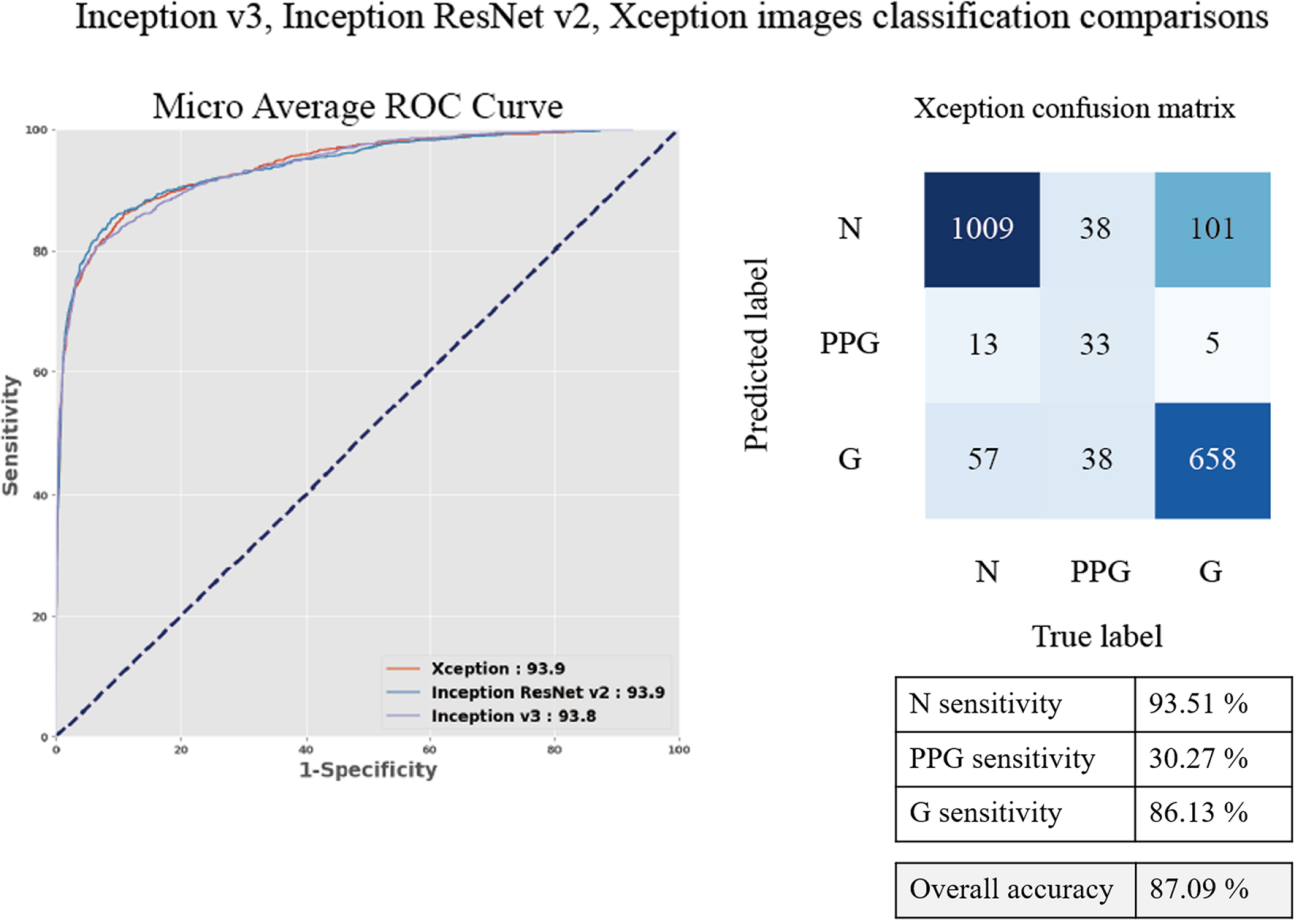
Inception v3, Inception ResNet v2, and Xception model comparison. Three CNN models: the Xception, Inception v3, and Inception ResNet v2 were compared. All of the models can achieve a 94% micro-average AUROC with tenfold cross-validation. Only the Xception model confusion matrix is shown because the model is the latest version of the three. Although the overall accuracy can reach 87.09%, the sensitivity for the PPG group was low. Only 33 of 109 images were correctly predicted as being PPG. CNN, convolutional neural network; AUROC, area under the receiver operating characteristic curve; PPG, pre-perimetric glaucoma

The results of selecting best base models for transfer learning from Xception, Inception v3, and Inception ResNet v2 were as shown in Fig. 3; there was no significant difference between these three CNN models. Although the overall accuracy of the model reached 87.09%, the PPG group's sensitivity was low. As shown in Fig. 3, out of 109 images, only 33 were correctly predicted as PPG (30.3% sensitivity). The Xception model was applied as the base model because it is the latest version of the inception network series. After collapsing the three groups into binary groups, the Xception model performance of the N, PPG + G groups reached 95.91% AUROC, 89.93% sensitivity, and 87.92% precision in the validation set. In the test set with the same grouping method, the model reached an AUROC of 95.45%, sensitivity of 88.88%, and precision of 96.53%. In the N + PPG, G groups, the model performance reached 96.72% AUROC, 90.54% sensitivity, and 94.13% precision with the validation set. With the test set, the performance reached 95.24% AUROC, 86.77% sensitivity, and 95.46% precision, as shown in Table 2. The N + PPG, G groups had a higher AUROC than the N, PPG + G groups for the validation set. Although the test results showed higher performance in the N, PPG + G groups, the increase was less than 1%, as shown in Table 2.

**Table 2:** Binary classification results after the three groups are collapsed into binary groups

Metrics	Validation dataset		Test dataset	
	(N^a^, PPG^b^ + G^c^)	(N^a^ + PPG^b^, G^c^)	(N^a^, PPG^b^ + G^c^)	(N^a^ + PPG^b^, G^c^)
AUROC^d^, %	95.91	96.42^e^	95.45^f^	95.24
Accuracy, %	89.96	90.57^e^	88.88^f^	86.11
Sensitivity, %	89.93	90.54^e^	88.63^f^	86.77
Specificity, %	89.99	90.59^e^	89.17^f^	85.49
Precision, %	87.92	94.13^e^	96.53^f^	95.46
F3^g^, %	89.73	89.80^e^	88.77^f^	86.56

^a^N: normal

^b^PPG: pre-perimetrical glaucoma

^c^G: glaucoma

^d^AUROC: area under the receiver operator characteristic curve

^e^best metrics value in the validation set

^f^best metrics value in the test set

^g^F3: F-Beta measure, beta = 3

### Regression model for predicting the OCTA-acquired cup/disc vertical ratio and average RNFL thickness

**Fig. 4:**
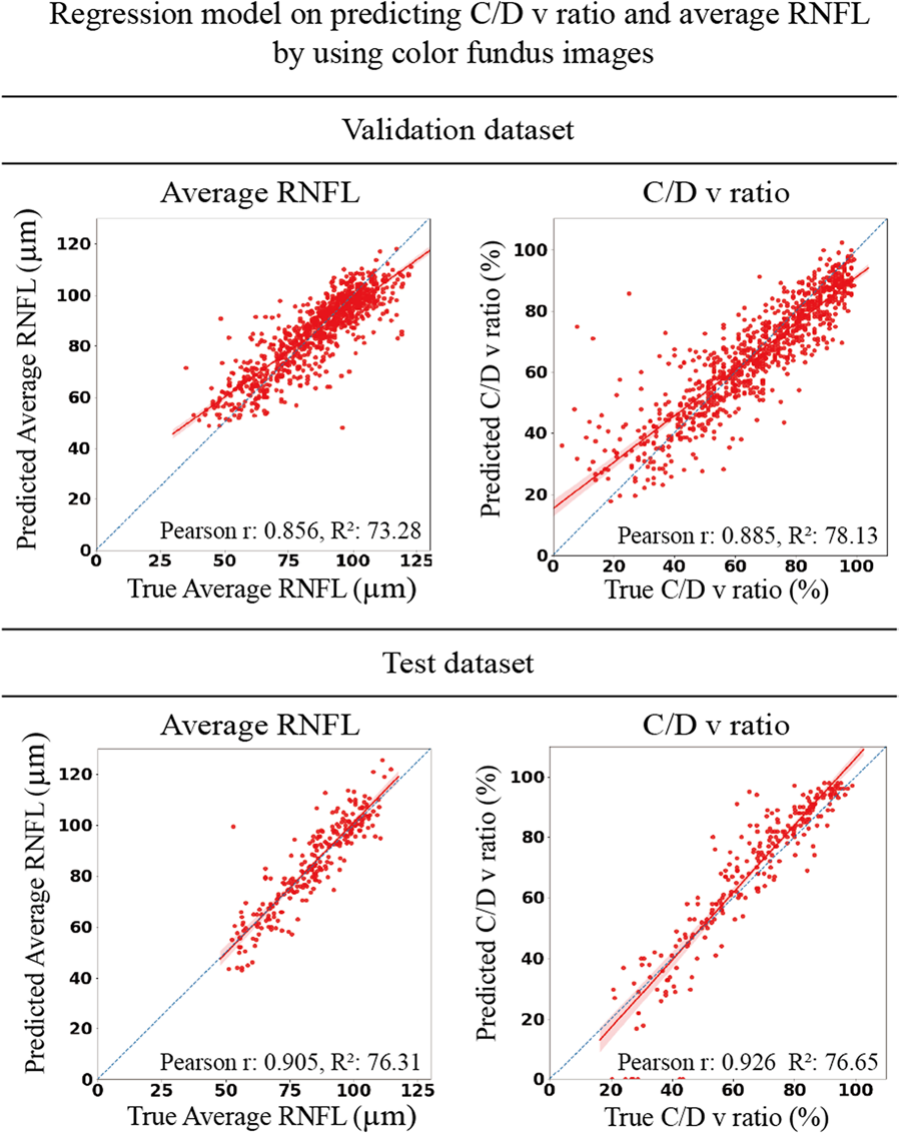
Regression model for predicting the OCTA acquired Cup/Disc vertical ratio and average RNFLT. The regression model can be trained to predict the average RNFLT and C/D V ratio from color fundus images (Pearson’s correlation coefficients: average RNFLT, 0.856; C/D V ratio, 0.885; R2: average RNFLT, 73.29%, Cup/Disc vertical ratio, 78.126). The test set showed similar results compared with the validation set. C/D V ratio, Cup/Disc vertical ratio; RNFLT, retinal nerve fiber layer thickness

The regression model was trained to predict the average RNFL thickness using the color fundus images. In the validation set, Pearson's correlation coefficient was 0.856, and the coefficient of determination (R^2^) was 73.29%. Moreover, as shown in Fig. 4, the model predicted the C/D v ratio with a high Pearson's correlation coefficient of 0.885 and an R^2^ of 78.13%. The model could also predict well in a test set with a high Pearson correlation coefficient (average RNFL: 0.905, C/D v ratio: 0.926) and R^2^ (average RNFL: 76.31%, C/D v ratio: 76.65%).

### Multimodal model

**Fig. 5:**
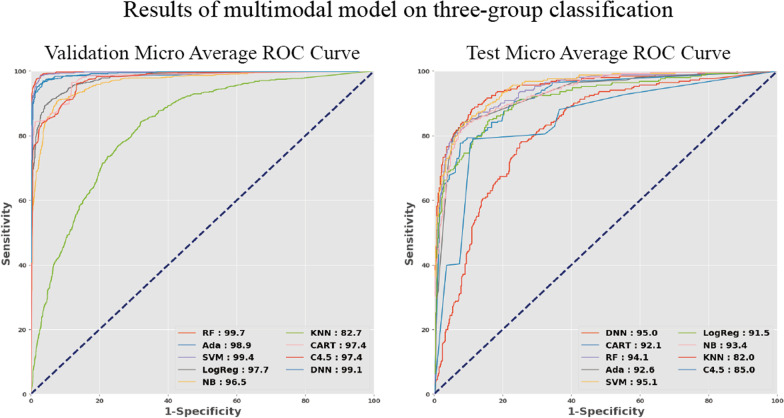
The micro average ROC of N, PPG, and G groups classified by multimodal models. Multimodal models achieved high AUROCs with the validation data. The RF (99.7%), SVM (99.4%), and DNN (99.1%) models showed similar results with tenfold cross-validation data. The best three models in the test set were the SVM (95.1%), DNN (95.0%), and RF (94.1%). There was a decrease in AUROCs in the test set, but they were still within an acceptable range. N, normal; G, glaucoma; RF, random forest; Ada, adaptive boosting; SVM, support vector machine; LogReg, logistic regression; NB, Naïve Bayes; KNN, k-nearest neighbor; CART: classification and regression decision tree; C4.5, C4.5 decision tree; DNN, dense neural network

**Fig. 6:**
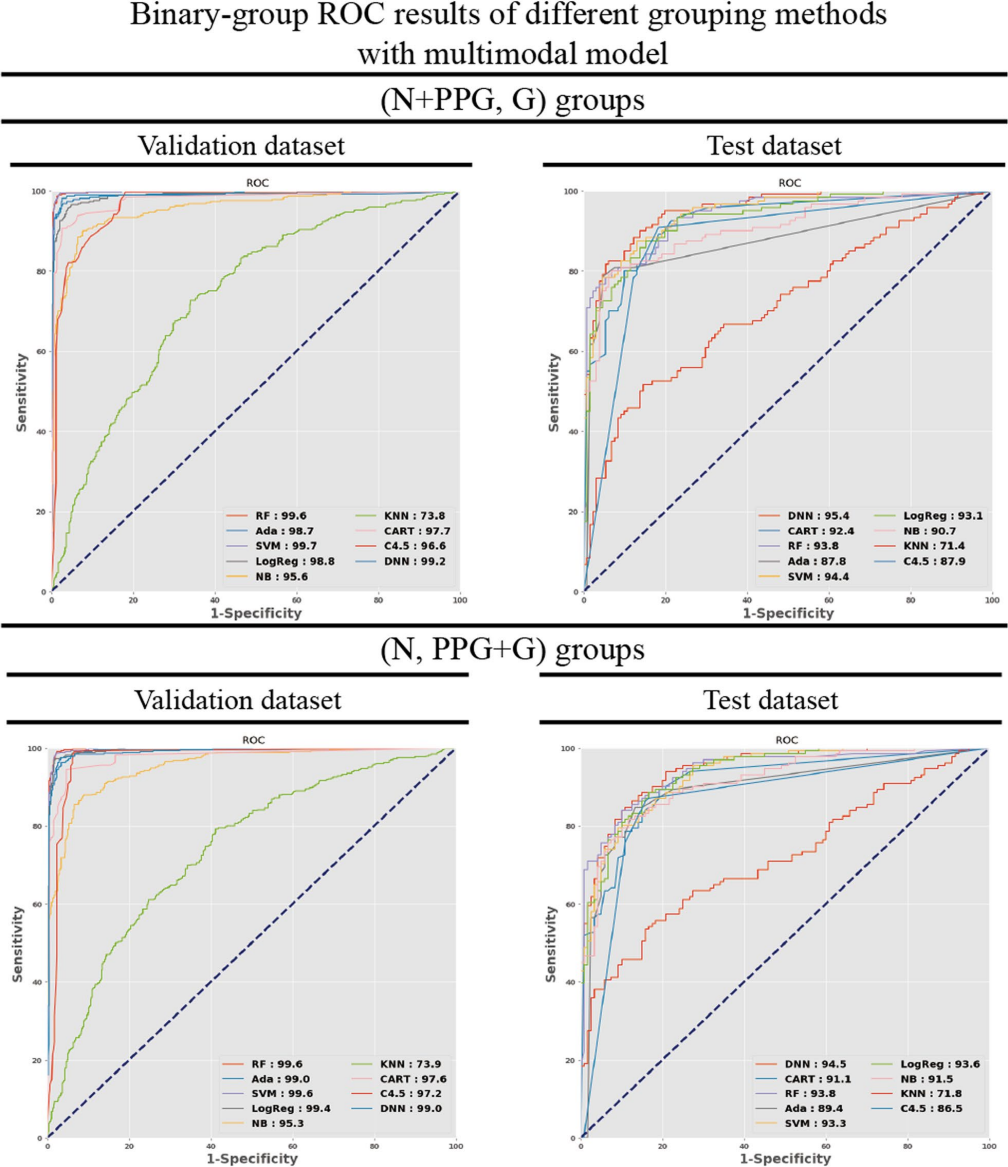
Binary-group classification results of multimodal models after applying different grouping strategies. RF, SVM, and DNN AUROCs were slightly higher in (N + PPG, G) groups (RF: 99.6%, SVM: 99.7%, DNN: 99.2%) and in (N, PPG + G) groups (RF: 99.6%, SVM: 99.6%, DNN: 99.0%) with tenfold cross validation. A similar result was shown in the test set in (N + PPG, G) groups (AUROCs: RF: 93.8%, SVM: 94.4%, DNN: 95.4%), and in (N, PPG + G) (AUROCs: RF: 93.8%, SVM: 93.3%, DNN: 94.5%). There were decreases in the AUROCs for the test dataset with both methods, but the best AUROC of the three models still exceeds 90%. RF, random forest; Ada, adaptive boosting; SVM, support vector machine; LogReg, logistic regression; NB, Naïve Bayes; KNN, k-nearest neighbor; CART, classification and regression tree; C4.5, C4.5 decision tree; DNN, dense neural network; AUROC, area under receiver operating characteristic curve

After predicting the C/D v ratio and average RNFL thickness using the regression model, the patients' demographic data and images were used to expand the model modalities. For a clear view of the multimodal models' performances, the top three models used in the final prediction were identified; the models' performances were shown in Figs. 5 and 6, and Additional file 5. In the validation and test sets, the top three models for classifying the N, PPG, and G groups were random forest, SVM, and DNN. These three models achieved high AUROCs in the validation set: random forest (99.7%), SVM (99.4%), and DNN (99.1%). In the test set, the AUROCs of the top three models were also high: SVM (95.1%), DNN (95.0%), and random forest (94.1%), as shown in Fig. 5.

The three groups were collapsed into binary groups in the multimodal models with the same rationale mentioned above. After converting the three-group classification to binary-group classification, the model performance improved. After the three groups were collapsed into binary groups, the multimodal models with the top three AUROCs remained the same as with the three-group classification (shown in Fig. 5), namely random forest, SVM, and DNN. In the N, PPG + G groups, the AUROCs of the random forest, SVM, and DNN models reached 99.56%, 99.59%, and 99.18% in the validation set. In the test set, the AUROCs slightly decreased to 93.77%, 94.42%, and 94.45%, respectively.

In contrast, in the (N + PPG, G groups, the AUROCs of the random forest, SVM, and DNN models reached 99.62%, 99.68%, and 99.01% in the validation set, and the AUROCs decreased slightly to 93.84%, 93.29%, and 95.38%, respectively, in the test set, as shown in Additional file 5 and Fig. 6. When comparing both grouping methods, the (N + PPG, G) groups showed the highest AUROCs in both the validation and test sets. The accuracy, precision, sensitivity, and F3 were computed after selecting the optimal cutoff point with Youden’s J statistic. These metrics showed a trend similar to the AUROCs for the top three models in both the test and validation sets, as shown in Additional file 5.

## Discussion

### Main findings

In this study, the multimodal model achieved an AUROC of 99.7% with SVM in the N + PPG, G groups in the validation set. The multimodal model using DNN maintained an AUROC of 95.4% in the test set, which showed promising results when detecting glaucoma in a myopic population. These improved performances were mainly attributed to the precise glaucoma grading of color fundus images with OCT extracted numerical data and the increase in model modalities that combined numerical data and images.

### Precise grading of normal, PPG, and glaucoma groups with OCT extracted numerical data

Li et al. trained an algorithm using a dataset of 31,745 fundus images, achieving an AUC of 0.986 [12]. Christopher et al. reported an AUC in the range of 0.79–0.97 by training AI with 17,195 images [13]. With a total of 3,132 fundus images, Shibata et al. achieved an AUC of 0.965 [28]. Compared to previous studies, several new strategies were adopted to create a new perspective in our study. First, the exclusion criterion was stricter to avoid lower-quality images and erroneous estimation results. This study excluded cataracts patients because cataracts undermines the quality of color fundus images and contributes to the underestimation of retinal layer thickness [29]. Second, our grading system was more objective. In previous studies, the ground truth about glaucoma was usually obtained by human judication through color fundus photography. Subjective evaluation resulted in low reproducibility, and even experienced ophthalmologists could not provide a fair grading standard [4, 30, 31]. Therefore, a precise and objective grading system was constructed using OCTA to collect patients' constructive data, by referring to other clinical examination histories, and divided subjects into three groups: normal, PPG, and glaucoma. This classification system was the first to consider OCT numerical data extracted from OCTA and visual field as grading standards, which was applied to AI trained with color fundus photography. Third, myopia was compatible with the worldwide pervading trend. In the dataset, 455 of 1155 participants (39%) had high myopia with axial length ≥ 26 mm. The results in Additional file 6 show that after separating myopia from non-myopia in test dataset. Both (N, PPG + G) and (N + PPG, G) shown 2–5% less AUROCs when compared myopia group to non-myopia. Although high myopia leads to relatively lower AUC values as high myopia cases are difficult to differentiate using color fundus images, patients with high myopia were still included in preparing for the era of high myopia prevalence.

### The benefit of using a multimodal model and a different approach for handling multimodality problems

Xception model was selected as the transfer learning base model although it showed similar performance with Inception v3 and Inception ResNet v2 mainly because it is the latest modification in the Inception model series [32–34]. Taking advantage of the Xception model and adopting it as a feature extractor in the proposed multimodal model, we proposed a multimodal model based on the following observations.

First, increasing the modalities often enhances the model's performance, which has been widely reported in the literature [35, 36]. However, few studies have increased the multimodality relevant to glaucoma diagnosis in machine learning. One example was the use of four different OCT images and color fundus images for glaucoma diagnosis through machine learning [37]. Second, to increase model accuracy when dealing with patients with high myopia, the values of axial length and visual acuity need to be included in the model's input. Third, multiple sources of information from various medical reports were viewed by experts before making a diagnosis of glaucoma.

The goal of this study was to make the model accessible in areas with low of medical resources. Therefore, OCT extracted numerical data were not included because the corresponding machines used to capture the data were expensive. To compensate for the lack of OCT extracted numerical data input in the multimodal model, a regression model was trained to predict the C/D v ratio and average RNFL thickness (which could be derived from the acquired OCT data) using color fundus images.

### The rationale for collapsed three groups into binary groups

The models in this study predicted N, PPG, and G's probabilities with each participant's data. The N + PPG, G groups have been commonly applied for precise G prediction in some studies. In contrast, grouping N, PPG + G would overestimate the G group. Whether PPG patients need treatment is still under debate. Studies suggested in PPG eyes, the mean defect progressions of VF were − 0.09 ± 0.25 [38], − 0.17 ± 0.72 [39], and − 0.39 ± 0.64 dB/year [40], respectively, slightly more severe than the median mean deviation rate of the population, − 0.05 dB/year [41]. In conclusion, which way to binarize the data is better requiring more research to rationalize.

### Differences between the test and validation sets

The multimodal model showed a slight decrease of almost 5% in AUROC for the test set compared to the training and validation sets. This decrease may be due to the following facts. First, the test set was collected in the last two weeks, while ten-fold cross-validation was performed in the first eight weeks. This difference in the time span is likely to degrade the performance of the test set. Second, the change in the PPG ratio over the total images in the test set may also degrade the performance. Although the test set did not perform as well as the validation set, it still achieved an AUROC of 95%, which is a good result compared with other studies that involved a large population of high myopia. As shown in Additional file 7, there are no significant differences between training and test sets in terms of age, axial length, HR, SBP, DBP, and visual acuity.

### Error analysis: false-positive and false-negative cases

The figure in Additional file 8 shows false-positive and false-negative cases by our model. Although our model achieved high AUROC with a high myopic population dataset, high myopic cases were still the majority in the false-positive cases, as shown in Additional file 8. On the other hand, false-negative cases show that our model would predict incorrectly with smaller disc, cup areas than normal color fundus images; these small cup and disc cases might have misleading cups-to-disc ratio and would cause the model to predict false-negative cases [42].

### Comparison with currently used methods and public datasets testing

The comparison table of our research and existing studies was shown in Additional file 9. Most of the listed studies trained the convolutional neural networks with single modalities (color fundus). Guangzhou An et al. study used a multimodal model/ ensemble learning model with OCTA and color fundus data and had a similar result with our model [35]. Although some studies showed higher test results than our study, these might cost by the high incidence of myopia in our study, or the limitations listed below.

To validate the model performance with external datasets, two Kaggle datasets were tested and evaluated [43, 44]. The datasets had different modalities compared to ours; therefore, a new pre-processing method and disc-centered model were used to compare the results. The methods and results were shown in Additional file 10. Additional file 10 showed that after retraining the disc-centered model with our training dataset and Kaggle training dataset (adapted model), tenfold cross-validation AUROC decreased by 3%–4% to 90.52% when compared to our original model with AUROC of 95.91% as shown in Table 2.

After testing the adapted model with the Kaggle test set, the AUROC dropped further to 68.88%, as shown in Additional file 11. The published codes on the Kaggle websites showed the same results as our model, which had approximately 60%–70% accuracy [45, 46]. To validate the code mentioned above, a tenfold cross-validation model was trained with only the Kaggle training dataset (model 2), as shown in Additional file 11. Our test set performed better than the Kaggle test set with AUROCs 86.61% and 70.47%, respectively, as shown in Additional file 11. The adaptation model mentioned above was applied and predicted using a second Kaggle dataset [44]. The second dataset had a large population but consisted of different types of ocular diseases. The color fundus images labeled as glaucoma (n = 203) and random normal fundus images (n = 300) were selected. Although the second Kaggle dataset had a higher AUROC of 83.01%, the first dataset still performed the worst.

We suspected that the main reason for the low performance of the first Kaggle dataset was mainly due to mismatch and several hypothesized reasons as below. First, the color fundus images acquired in these two datasets might have been collected from different populations. Second, the fundus of false-positive cases showed several images that our dataset had excluded, such as blurred images that might have been caused by cataracts and other systemic disorders, as shown in Additional file 11. Third, the imbalance ratios in the two datasets were different. The Kaggle dataset collected more normal images. The imbalance of data in machine learning is still an obstacle that is difficult to overcome. For these reasons, it is important that the user strictly follow the guidelines listed on the web page when uploading their data to predict an optimal result.

### Limitations

One limitation of this study is its relatively small database. However, the database contains 1,150 color fundus photographs, complete ophthalmological data (for example, OCTA, visual field, IOP, axial length), and an accurate grading system. If more data are collected, the algorithm might achieve a higher AUROC value.

Undoubtedly, the major limitation of this study is that some potential confounders such as age and axial length were not distributed equally within groups. Young adults showed a higher incidence of myopia. We also adopted some methods to control for confounding effects, such as the exclusion of cataracts and age-related macular disease, and a questionnaire about participants’ health condition was completed beforehand. A better sampling method is difficult, but is expected in our future work.

With the proposed multimodal model, large-scale screening and telemedicine can be applied in rural areas. Moreover, a webpage was built to establish a telemedicine website using the multimodal model [47]. We would like to release labeled color fundus data for interested researchers and academic studies. An example of a labeled image was presented in Additional file 12. The model and data will be free to use and can be easily accessed globally.

## Supplementary Information

**Additional file 1**. Images preprocessing procedure.

**Additional file 2**. Glaucoma decision support system and web application flow chart.

**Additional file 3**. Evaluation metrics equations.

**Additional file 4**. The correlation between features and outcomes.

**Additional file 5.** The results comparison of multimodal models. **Additional File Table 1.** Comparison of all model performances in the validation set (Na, PPGb+Gc) groups. **Additional File Table 2.** Comparison of all model performances in the validation set (Na + PPGb, Gc) groups. **Additional File Table 3.** Comparison of all model performances in the test set (Na, PPGb+Gc) groups. **Additional File Table 4.** Comparison of all model performances in the test set (Na + PPGb, Gc) groups.

**Additional file 6**. Comparison of non-myopia and myopia AUROCs in test datasets.

**Additional file 7.** The characteristics of testing and training sets. **Additional File 7 Table 5.** Descriptive statistics on training-validation and test datasets. **Additional File 7 Figure 1.** Training-validation and testing datasets used features box-whisker and violin plots.

**Additional file 8**. Error analysis on our model false-positive and false-negative cases. 

**Additional file 9**. Comparison with currently used methods. **Additional File Table 6**, Comparison with currently used methods.

**Additional file 10**. Pre-processing method for public datasets testing and training results. **Additional File Table 7.** The 10-fold cross validation results of Kaggle public datasets.

**Additional file 11**. Publics dataset testing results and error analysis. **Additional File Table 8.** The test results of different Kaggle datasets with different models.

**Additional file 12**. Glaucoma feature labeling tools and guidelines.

## Data Availability

The datasets used and/or analyzed during the current study and the labelling dataset mentioned in Additional file 12 are available from the corresponding author upon reasonable request.

## Abbreviations

Ada: Adaptive-boost
AI: Artificial intelligence
AUROC: Area under receiver operator characteristic curve
CART: Classification and regression tree decision tree
C/D v ratio: Cup/disc vertical ratio
CNN: Convolutional neural network
DNN: Dense neural network
G: Glaucoma
IOP: Intraocular pressure
KNN: K-nearest neighbors
LogReg: Logistic regression
N: Normal
NB: Naïve Bayes
OCTA: Optical coherence tomography angiography
PPG: Pre-perimetric glaucoma
RF: Random forest
RNFL: Retinal nerve fiber layer
SVM: Support vector machine
