# Supplementary material for: Use of multimodal dataset in AI for detecting glaucoma based on fundus photographs assessed with OCT: focus group study on high prevalence of myopia
Source: BMC Med Imaging. 2022 Nov 24;22:206. doi: 10.1186/s12880-022-00933-z (PMC9700928; doi:10.1186/s12880-022-00933-z)

### Additional File 1: Images preprocessing

First, the color fundus images were binarized. The largest outer contour on the images, which is the optic camera's outer border, was detected by the border following the algorithm provided by the A minimally enclosed circle was drawn from the acquired contour and used for cropping the image. After removing the areas, the images were resized to 299 × 299 pixels, with the pixel value from 0 to 1. Proper augmentations, such as horizontal and vertical flips and image rotation, were applied to increase training images.


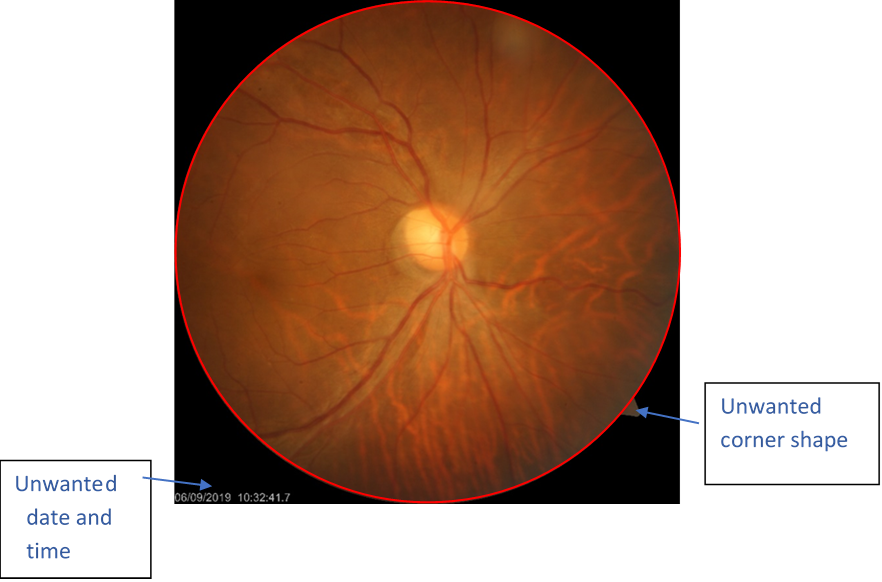

Supplement: Supplementary file 1 — Additional file 1. Images preprocessing procedure. [file 12880_2022_933_MOESM1_ESM.docx]
