# Supplementary material for: Use of multimodal dataset in AI for detecting glaucoma based on fundus photographs assessed with OCT: focus group study on high prevalence of myopia
Source: BMC Med Imaging. 2022 Nov 24;22:206. doi: 10.1186/s12880-022-00933-z (PMC9700928; doi:10.1186/s12880-022-00933-z)
Supplement: Supplementary file 2 — Additional file 2. Glaucoma decision support system and web application flow chart. [file 12880_2022_933_MOESM2_ESM.docx]

### Additional File 2: Glaucoma decision support system and web application flow chart


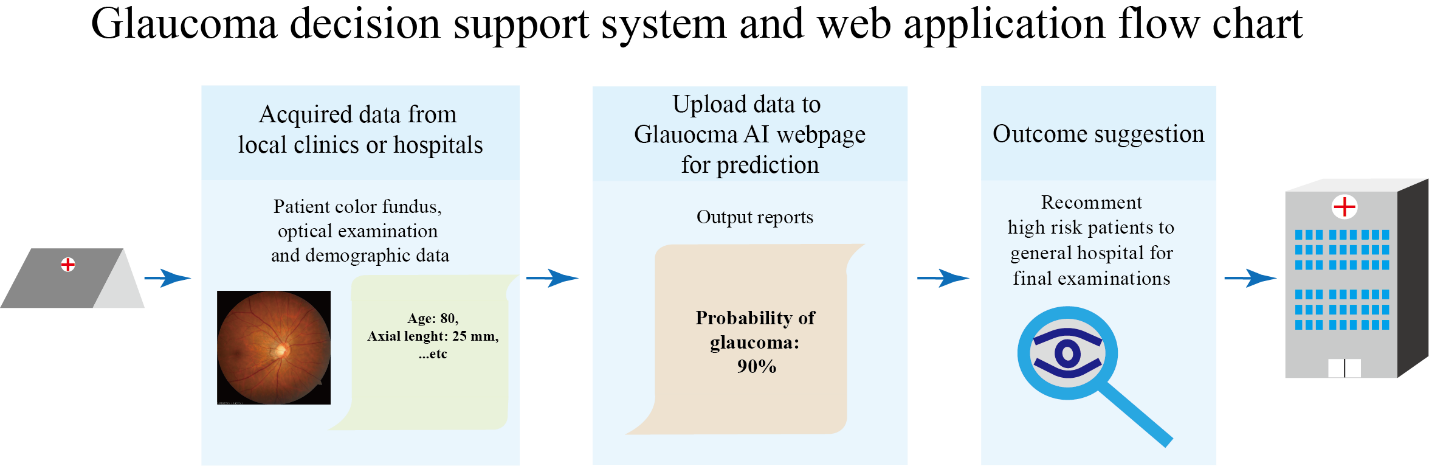


A webpage was designed for the decision support system. The webpage can be accessed by anyone who has internet access. They can upload the color fundus they acquired with optic examination and patients’ demographic data from local clinics or hospitals to our server. Users were required to follow the guidelines when uploading their data, the units and range of the numerical inputs were given to prevent inaccurate upload, and the 45-degree fundus images must have a minimum 1500 x 1500 resolution. Two fundus images examples and all the guidelines were listed on the webpage. The model will predict the outcome, and a result webpage will generate for the users and suggest high-risk patients go to the general hospital to follow up medical checking.
