# Supplementary material for: Use of multimodal dataset in AI for detecting glaucoma based on fundus photographs assessed with OCT: focus group study on high prevalence of myopia
Source: BMC Med Imaging. 2022 Nov 24;22:206. doi: 10.1186/s12880-022-00933-z (PMC9700928; doi:10.1186/s12880-022-00933-z)
Supplement: Supplementary file 3 — Additional file 3. Evaluation metrics equations. [file 12880_2022_933_MOESM3_ESM.docx]

### Additional File 3: Evaluation metrics equations

$Precision= \frac{tp}{tp +fp}$ (1)

$Sensitivity = \frac{tp}{tp+fn}$ (2)

$Accuracy= \frac{tp+tn}{tp+fp+tn+fn}$ (3)

$Specificity = \frac{tn}{tn +fp}$ (4)

$F_{\beta} = \left( 1+\beta^{2} \right)\cdot\frac{precision \cdot sensitivity}{\left( \beta^{2} \cdot precision \right) + sensitivity}$ (5)

$Youden's J statisrtic = sensitivity +specificity - 1$ (6)
