# Supplementary material for: Use of multimodal dataset in AI for detecting glaucoma based on fundus photographs assessed with OCT: focus group study on high prevalence of myopia
Source: BMC Med Imaging. 2022 Nov 24;22:206. doi: 10.1186/s12880-022-00933-z (PMC9700928; doi:10.1186/s12880-022-00933-z)
Supplement: Supplementary file 4 — Additional file 4. The correlation between features and outcomes. [file 12880_2022_933_MOESM4_ESM.docx]

### Additional File 4: The correlation between features and outcomes.


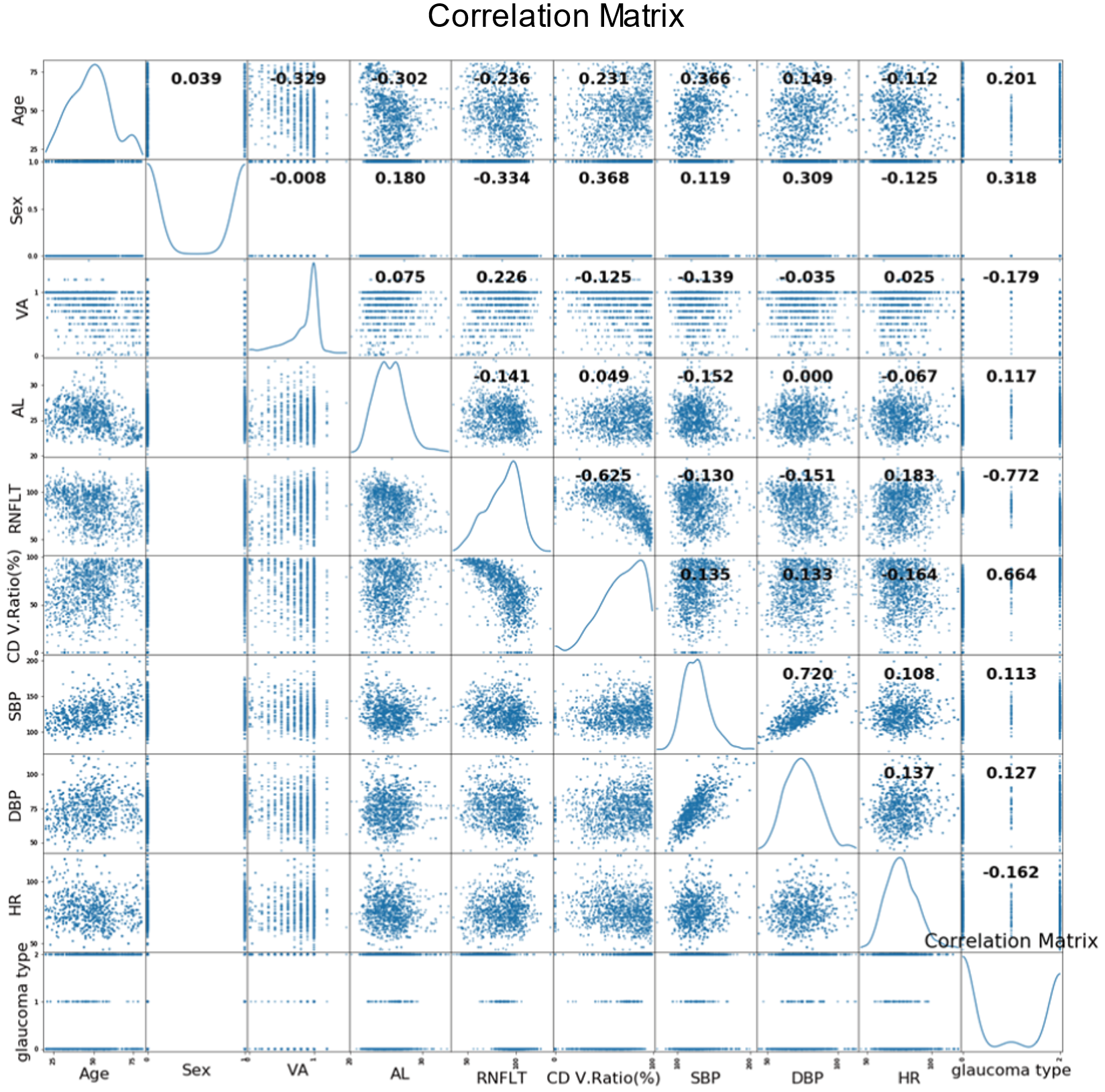


The correlation between two continuous variables was calculated with Pearson’s correlation, and correlation between continuous and categorical variables was calculated with point-biserial correlation. The RNFLT (-0.772) and CD v ratio (0.66) had a high correlation with our outcome glaucoma type. The correlation matrix did not show other variables that had a high correlation with the predictors and outcomes. We suggest the potential confounders were age, sex, axial length, visual acuity, heart rate, and blood pressure; and suggest that the effect modifiers were C/D v ratio and RNFLT.
