# Supplementary material for: Use of multimodal dataset in AI for detecting glaucoma based on fundus photographs assessed with OCT: focus group study on high prevalence of myopia
Source: BMC Med Imaging. 2022 Nov 24;22:206. doi: 10.1186/s12880-022-00933-z (PMC9700928; doi:10.1186/s12880-022-00933-z)
Supplement: Supplementary file 5 — Additional file 5. The results comparison of multimodal models. Additional File Table 1. Comparison of all model performances in the validation set (Na, PPGb+Gc) groups. Additional File Table 2. Comparison of all model performances in the validation set (Na + PPGb, Gc) groups. Additional File Table 3. Comparison of all model performances in the test set (Na, PPGb+Gc) groups. Additional File Table 4. Comparison of all model performances in the test set (Na + PPGb, Gc) groups. [file 12880_2022_933_MOESM5_ESM.docx]

### Additional File 5: The results comparison of multimodal models

| **Additional File Table 1. Comparison of all model performances in the validation set (N^a^, PPG^b^+G^c^) groups** | | | | | | | | | | |
| --- | --- | --- | --- | --- | --- | --- | --- | --- | --- | --- |
| Metrics | Multimodal Models | | | | | | | | | Classification Model |
|  | RF^d^ | Ada^e^ | SVM^f^ | LogReg^g^ | NB^h^ | KNN^i^ | CART^j^ | C4.5^k^ | DNN^l^ | CNN^m^ |
| **AUROC^n^, %** | 99.62 | 98.69 | 99.68^o^ | 98.81 | 95.58 | 73.77 | 97.66 | 96.62 | 99.18 | 95.91 |
| **Accuracy, %** | 98.67^o^ | 97.35 | 98.34 | 95.69 | 90.82 | 68.36 | 93.69 | 89.49 | 97.01 | 89.96 |
| **Specificity, %** | 98.13^o^ | 96.29 | 97.61 | 93.95 | 87.56 | 60.28 | 91.15 | 86.01 | 96.01 | 89.99 |
| **Sensitivity, %** | 98.66^o^ | 97.32 | 98.39 | 95.71 | 90.62 | 68.36 | 93.83 | 89.01 | 96.78 | 89.93 |
| **F3^p^, %** | 98.61^o^ | 97.21 | 98.31 | 95.53 | 90.30 | 67.46 | 93.56 | 88.70 | 96.71 | 89.73 |
| ^a^N: normal,  ^b^PPG: pre-perimetrical glaucoma,  ^c^G: glaucoma,  ^d^RF: random forest  ^e^Ada: Ada-boost  ^f^SVM: support vector machine  ^g^LogReg: logistic regression  ^h^NB: Naïve Bayes  ^i^KNN: k-nearest neighbor  ^j^CART: classification and regression tree decision tree  ^k^C4.5: C4.5 algorithm decision tree  ^l^DNN: dense neural network  ^m^CNN: convolutional neural network  ^n^AUROC: area under receiver operating characteristic curve  ^o^: best metric results  ^p^F3: F-beta measure (beta=3) | | | | | | | | | | |

| **Additional File Table 2. Comparison of all model performances in the validation set (N^a^ + PPG^b^, G^c^) groups** | | | | | | | | | | |
| --- | --- | --- | --- | --- | --- | --- | --- | --- | --- | --- |
| Metrics (%) | Multimodal Models | | | | | | | | | Classification Model |
|  | RF^d^ | Ada^e^ | SVM^f^ | LogReg^g^ | NB^h^ | KNN^i^ | CART^j^ | C4.5^k^ | DNN^l^ | CNN^m^ |
| **AUROC^n^, %** | 99.56 | 99.00 | 99.59^o^ | 99.44 | 95.32 | 73.89 | 97.59 | 97.24 | 99.01 | 96.42 |
| **Accuracy, %** | 98.12^o^ | 96.35 | 98.12^o^ | 97.01 | 88.05 | 67.04 | 94.91 | 94.47 | 97.01 | 90.57 |
| **Specificity, %** | 97.90^o^ | 96.04 | 97.90^o^ | 96.52 | 86.87 | 64.64 | 94.84 | 93.75 | 96.74 | 90.59 |
| **Sensitivity, %** | 98.13^o^ | 96.26 | 98.13^o^ | 97.20 | 88.08 | 67.06 | 94.39 | 94.63 | 96.96 | 90.54 |
| **F3^p^, %** | 98.11^o^ | 96.24 | 98.11^o^ | 97.13 | 87.96 | 66.81 | 94.44 | 94.54 | 96.94 | 89.80 |
| ^a^N: normal,  ^b^PPG: pre-perimetrical glaucoma,  ^c^G: glaucoma,  ^d^RF: random forest  ^e^Ada: Ada-boost  ^f^SVM: support vector machine  ^g^LogReg: logistic regression  ^h^NB: Naïve Bayes  ^i^KNN: k-nearest neighbor  ^j^CART: classification and regression tree decision tree  ^k^C4.5: C4.5 algorithm decision tree  ^l^DNN: dense neural network  ^m^CNN: convolutional neural network  ^n^AUROC: area under receiver operating characteristic curve  ^o^: best metric results  ^p^F3: F-beta measure (beta=3) | | | | | | | | | | |

| **Additional File Table 3. Comparison of all model performances in the test set (N^a^, PPG^b^+G^c^) groups** | | | | | | | | | | |
| --- | --- | --- | --- | --- | --- | --- | --- | --- | --- | --- |
| Metrics (%) | Multimodal Models | | | | | | | | | Classification Model |
|  | RF^d^ | Ada^e^ | SVM^f^ | LogReg^g^ | NB^h^ | KNN^i^ | CART^j^ | C4.5^k^ | DNN^l^ | CNN^m^ |
| **AUROC^n^, %** | 93.84 | 89.35 | 93.29 | 93.58 | 91.47 | 71.77 | 91.13 | 86.51 | 94.45 | 95.24^o^ |
| **Accuracy, %** | 85.66 | 84.86 | 84.06 | 85.66 | 83.27 | 65.74 | 84.06 | 85.26 | 86.06 | 88.88^o^ |
| **Specificity, %** | 86.82 | 86.05 | 85.27 | 86.26 | 84.50 | 67.72 | 85.27 | 85.61 | 86.92 | 89.17^o^ |
| **Sensitivity, %** | 85.50 | 84.73 | 83.97 | 86.26 | 83.21 | 65.65 | 83.97 | 86.26 | 86.26 | 88.63^o^ |
| **F3^p^, %** | 85.63 | 84.86 | 84.10 | 86.26 | 83.33 | 65.85 | 84.10 | 86.19 | 86.33 | 88.77^o^ |
| ^a^N: normal,  ^b^PPG: pre-perimetrical glaucoma,  ^c^G: glaucoma,  ^d^RF: random forest  ^e^Ada: Ada-boost  ^f^SVM: support vector machine  ^g^LogReg: logistic regression  ^h^NB: Naïve Bayes  ^i^KNN: k-nearest neighbor  ^j^CART: classification and regression tree decision tree  ^k^C4.5: C4.5 algorithm decision tree  ^l^DNN: dense neural network  ^m^CNN: convolutional neural network  ^n^AUROC: area under receiver operating characteristic curve  ^o^: best metric results  ^p^F3: F-beta measure (beta=3) | | | | | | | | | | |

| **Additional File Table 4. Comparison of all model performances in the test set (N^a^ + PPG^b^, G^c^) groups** | | | | | | | | | | |
| --- | --- | --- | --- | --- | --- | --- | --- | --- | --- | --- |
| **Metrics (%)** | **Multimodal Models** | | | | | | | | | **Classification Model** |
|  | RF^d^ | Ada^e^ | SVM^f^ | LogReg^g^ | NB^h^ | KNN^i^ | CART^j^ | C4.5^k^ | DNN^l^ | CNN^m^ |
| **AUROC^n^, %** | 93.77 | 87.75 | 94.43 | 93.07 | 90.71 | 71.37 | 92.39 | 87.88 | 95.38^o^ | 95.24 |
| **Accuracy, %** | 84.46 | 84.46 | 87.25 | 86.06 | 82.07 | 65.74 | 83.27 | 83.67 | 88.05^o^ | 86.11 |
| **Specificity, %** | 83.47 | 85.84 | 86.07 | 85.12 | 80.49 | 63.71 | 82.50 | 84.96 | 86.89^o^ | 85.49 |
| **Sensitivity, %** | 84.17 | 80.83 | 87.50 | 85.83 | 82.50 | 65.83 | 82.50 | 80.00 | 88.33^o^ | 86.77 |
| **F3^p^, %** | 84.10 | 81.31 | 87.35 | 85.76 | 82.29 | 65.61 | 82.50 | 80.47 | 88.19^o^ | 86.56 |
| ^a^N: normal,  ^b^PPG: pre-perimetrical glaucoma,  ^c^G: glaucoma,  ^d^RF: random forest  ^e^Ada: Ada-boost  ^f^SVM: support vector machine  ^g^LogReg: logistic regression  ^h^NB: Naïve Bayes  ^i^KNN: k-nearest neighbor  ^j^CART: classification and regression tree decision tree  ^k^C4.5: C4.5 algorithm decision tree  ^l^DNN: dense neural network  ^m^CNN: convolutional neural network  ^n^AUROC: area under receiver operating characteristic curve  ^o^: best metric results  ^p^F3: F-beta measure (beta=3) | | | | | | | | | | |
