# Supplementary material for: Use of multimodal dataset in AI for detecting glaucoma based on fundus photographs assessed with OCT: focus group study on high prevalence of myopia
Source: BMC Med Imaging. 2022 Nov 24;22:206. doi: 10.1186/s12880-022-00933-z (PMC9700928; doi:10.1186/s12880-022-00933-z)
Supplement: Supplementary file 6 — Additional file 6. Comparison of non-myopia and myopia AUROCs in test datasets. [file 12880_2022_933_MOESM6_ESM.docx]

Additional File 6: Comparison of non-myopia and myopia AUROCs in test datasets

We separated non-myopia population from myopia population in test dataset. The evaluation on these two groups showed high myopia cost difficulty on glaucoma diagnosing, and lower the AUROCs when compared to non-myopia groups. The highest AUROC of non-myopia groups in (N, PPG+G) was DNN 96.1% and in (N+PPG, G) was also DNN with 95.9 %. The AUROCs of myopia groups showed 2-5% lower compared to non-myopia groups, with highest of DNN 90.4% in (N, PPG+G) and the highest in (N+PPG, G) was DNN 92.0%.


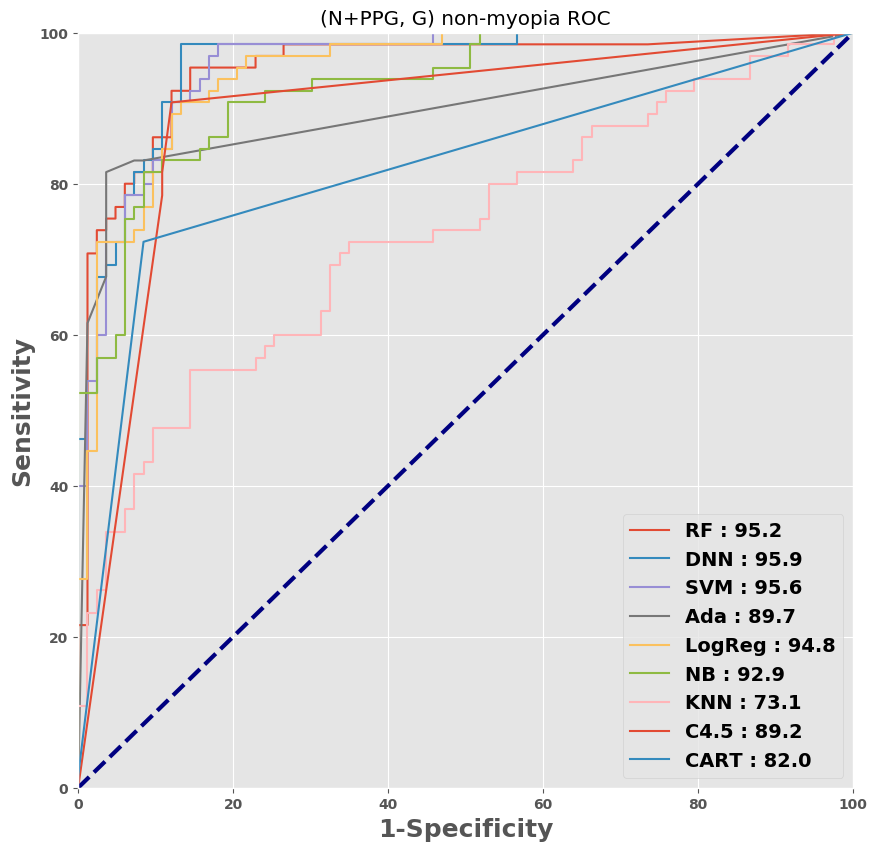

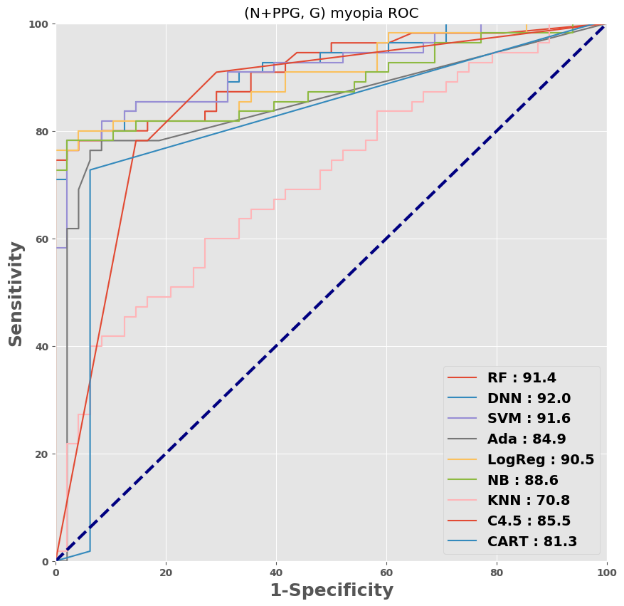

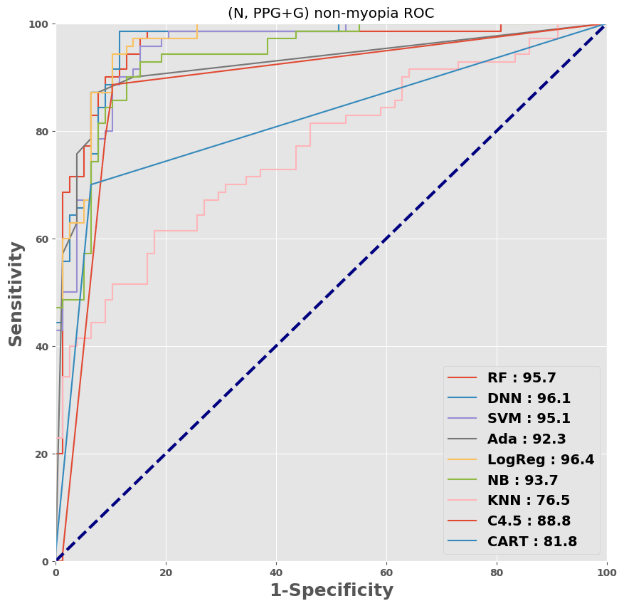

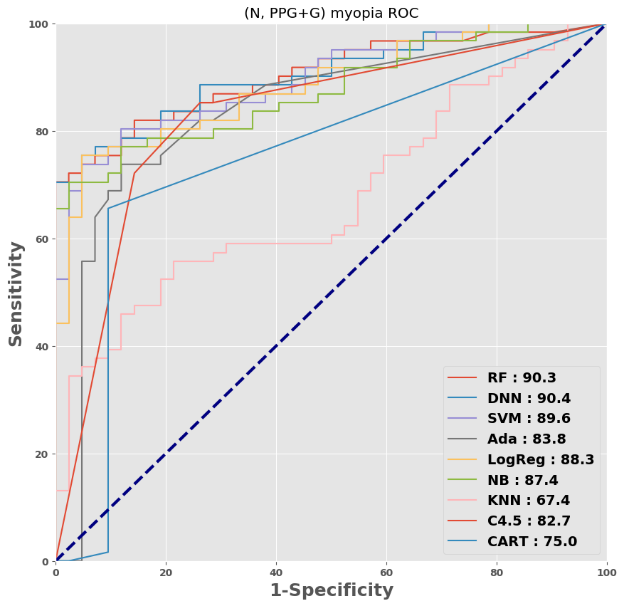


Comparison of non-myopia and myopia AUROCs in test dataset (N, PPG+G) groups

Comparison of non-myopia and myopia AUROCs in test dataset (N+PPG, G) groups
