# Supplementary material for: Use of multimodal dataset in AI for detecting glaucoma based on fundus photographs assessed with OCT: focus group study on high prevalence of myopia
Source: BMC Med Imaging. 2022 Nov 24;22:206. doi: 10.1186/s12880-022-00933-z (PMC9700928; doi:10.1186/s12880-022-00933-z)

**Additional File 7: The characteristics of testing and training sets**

The characteristics of testing and training sets are shown in Additional Tables 5. We only analyzed the features we used in training the models. There are no significant differences between training and test set age, axial length, HR, SBP, DBP, and visual acuity. However, test set patients show a little older population and had more male patients, but the differences were still within a tolerable range. To further demonstrate that our model can discriminate between glaucoma and high myopia, we also described the characteristics of average retinal nerve fiber layer (RNFL) and cup/disc vertical (CD V) ratio in control, PPG, and glaucoma. Table 5, boxplots, and violin plots showed that the average RNFL was significantly lower in glaucoma patients compared to controls, and the CD v ratio was significantly increased in glaucoma patients. The distributions of average RNFL and CD v ratios were different for control and glaucoma, as shown in the violin plot below. These findings may further suggest the importance of applying a regression model that predicts average RNFL and CD v ratios on fundus images, which were then used to train our multimodal model.

| **Additional File 7 Table 5. Descriptive statistics on training-validation and test datasets** | | | | | | | | |
| --- | --- | --- | --- | --- | --- | --- | --- | --- |
|  | Training set | | | Training set Control vs Glaucoma P-value | Test set | | | Test set Control vs Glaucoma P-value |
|  | Control | PPG^a^ | Glaucoma |  | Control | PPG^a^ | Glaucoma |  |
| Sex |  |  |  |  |  |  |  |  |
| Female, n | 316 | 20 | 137 |  | 70 | 3 | 32 |  |
| Male, n | 160 | 35 | 236 |  | 50 | 8 | 88 |  |
| Age, mean ± SD^b^ | 51.06 ± 13.66 | 44.92 ± 14.42 | 46.11 ± 13.04 | 4.81833E-10 | 53.56 ± 13.52 | 47.30 ± 13.34 | 42.36 ± 10.70 | 0.000375 |
| Axial length (mm),  mean ± SD^b^ | 25.64 ± 2.24 | 25.20 ± 1.77 | 25.93 ± 1.96 | 0.00171079 | 25.77 ± 2.16 | 25.30 ± 1.82 | 26.04 ± 1.28 | 0.074967 |
| HR^c^,  mean ± SD^b^ | 75.37 ± 12.15 | 79.15 ± 11.95 | 75.73 ± 10.15 | 6.51891E-06 | 74.76 ± 13.32 | 79.28 ± 13.37 | 71.27 ± 11.89 | 0.009177 |
| SBP^d^,  mean ± SD^b^ | 128.26 ± 18.07 | 123.92 ± 18.57 | 129.20 ± 16.91 | 0.000656205 | 128.98 ± 17.59 | 123.46 ± 16.45 | 124.00 ± 10.65 | 0.012758 |
| DBP^e^,  mean ± SD^b^ | 76.08 ± 12.09 | 73.27 ± 12.55 | 78.80 ± 12.79 | 0.001044251 | 76.83 ± 11.59 | 72.58 ± 12.00 | 76.00±9.85 | 0.005692 |
| Visual acuity (logMAR), mean ± SD^b^ | 0.79 ± 0.28 | 0.92 ± 0.14 | 0.85 ± 0.26 | 2.39484E-17 | 0.81 ± 0.28 | 0.90 ± 0.17 | 0.99 ± 0.03 | 0.002381 |
| CD V.ratio(%)_f_ ,mean ± SD^b^ | 53.96 ± 16.84 | 68.02 ± 16.27 | 82.53 ± 13.61 | 9.7753E-114 | 51.89 ± 20.94 | 77.45 ± 13.39 | 85.27 ± 11.56 | 6.679E-152 |
| Average RNFL^g^,  mean ± SD^b^ | 97.83 ± 8.87 | 83.85 ± 9.19 | 71.54 ± 14.42 | 3.33502E-37 | 99.35 ± 8.41 | 86.18 ± 5.86 | 69.75 ± 11.37 | 3.33545E-62 |
| ^a^PPG: pre-perimetric glaucoma  ^b^SD: Standard deviation  ^c^HR: Heart rate  ^d^SBP: Systolic blood pressure  ^e^DBP: Diastolic blood pressure  ^f^CD v ratio: cup/disc vertical ratio  ^g^RNFL: Retinal nerve fiber layer | | | | | | | | |

**Additional File 7 Figure 1. Training-validation and testing datasets used features box-whisker and violin plots.**


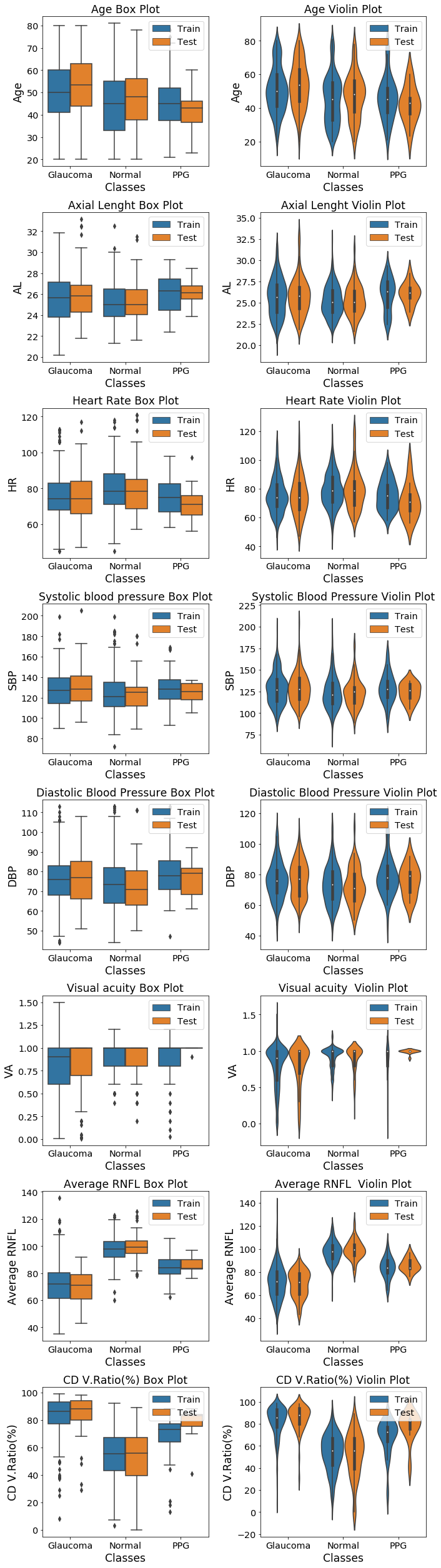


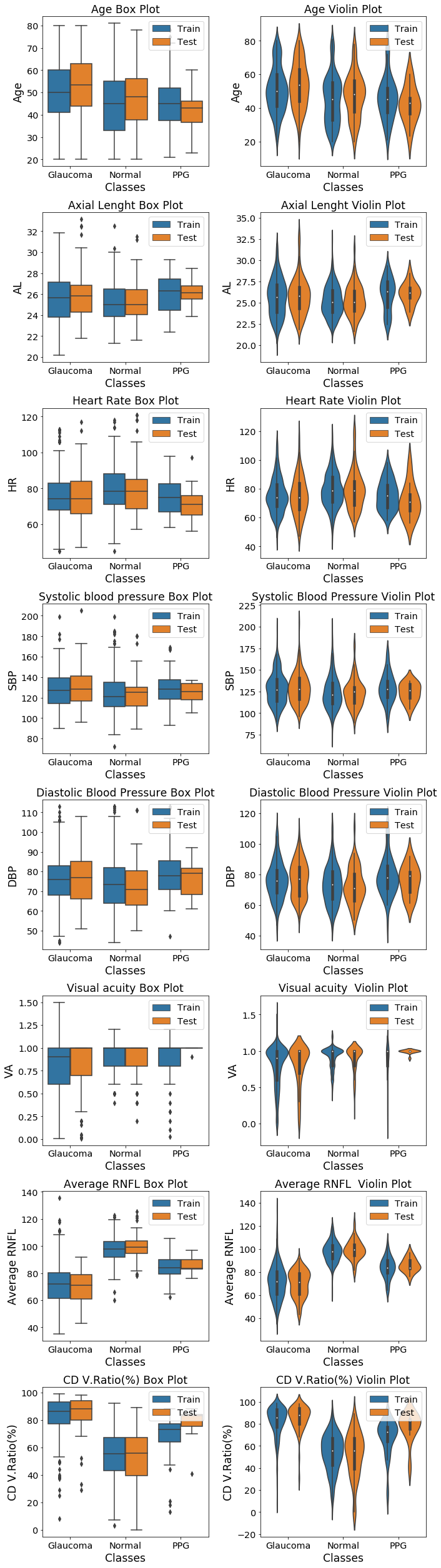

Supplement: Supplementary file 7 — Additional file 7. The characteristics of testing and training sets. Additional File 7 Table 5. Descriptive statistics on training-validation and test datasets. Additional File 7 Figure 1. Training-validation and testing datasets used features box-whisker and violin plots. [file 12880_2022_933_MOESM7_ESM.docx]
