# Supplementary figures and images for: Use of multimodal dataset in AI for detecting glaucoma based on fundus photographs assessed with OCT: focus group study on high prevalence of myopia
Source: BMC Med Imaging. 2022 Nov 24;22:206. doi: 10.1186/s12880-022-00933-z (PMC9700928; doi:10.1186/s12880-022-00933-z)

### Additional File 8: Error analysis on our model false-positive and false-negative cases


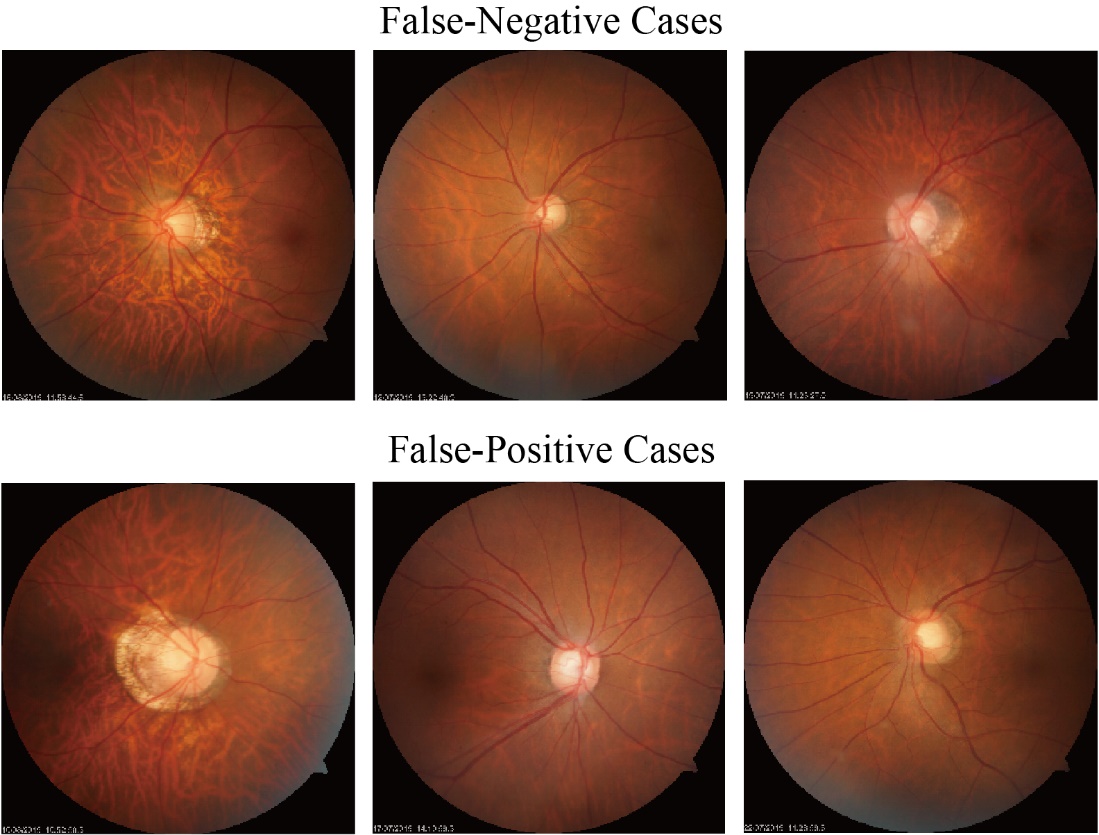

Supplement: Supplementary file 8 — Additional file 8. Error analysis on our model false-positive and false-negative cases. [file 12880_2022_933_MOESM8_ESM.docx]
