# Supplementary material for: Use of multimodal dataset in AI for detecting glaucoma based on fundus photographs assessed with OCT: focus group study on high prevalence of myopia
Source: BMC Med Imaging. 2022 Nov 24;22:206. doi: 10.1186/s12880-022-00933-z (PMC9700928; doi:10.1186/s12880-022-00933-z)
Supplement: Supplementary file 9 — Additional file 9. Comparison with currently used methods. Additional File Table 6, Comparison with currently used methods. [file 12880_2022_933_MOESM9_ESM.docx]

### Additional File 9: Comparison with currently used methods

| Additional File Table 6, Comparison with currently used methods | | | | | | | | | | |
| --- | --- | --- | --- | --- | --- | --- | --- | --- | --- | --- |
| Authors | Year | Model(s) used | Validation performance | | Testing performance | | Datasets (sample size) | | | Number of input modalities |
|  |  |  | AUROC^a^, % | Accuracy, % | AUROC^a^, % | Accuracy, % | Glaucoma | Normal | Others |  |
| Our study |  | Multimodal model (Xception as images features extractors) | 99.59 | 98.12 | 95.38 | 88.05 | 493 | 596 | 66 (PPG) | 2 (Numerical, Images) |
| Zhixi Li, et al. [1] | 2018/8/1 | Inception-v3 | 98.6 | - | - | - | 6,122 | 23,433 | 2,190 (suspect) | 1 (Images) |
| Mark Christopher [2] | 2018/10/1 | VGG16^b^, Inception V3, ResNet50^c^ | 91 | - | - | - | 5,633 | 9,189 | - | 1 (Images) |
| Lama A. Al-Aswad et al. [3] | 2019/12/1 | Pegasus (ResNet50^c^ based) | - | - | 92.6 | - | Training: 136,146 Testing: glaucoma:50, normal: 50 | | | 1 (Images) |
| Ruben Hemelings et al. [4] | 1905/7/12 | ResNet50 | - | - | 99.5 | - | 6,651 | 1,782 (1,614+168) | - | 1 (Images) |
| Sang Phan et al. [5] | 2019/2/24 | VGG19^b^, ResNet152^c^, DenseNet201 | - | - | 99.9-88.2 | - | 625 (369+256) | 2,687 | - | 1 (Images) |
| Naoto Shibata et al. [6] | 2018/10/2 | ResNet^c^ | 94.1-96.0 | - | 96.5 | - | 1,424 (1,364+60) | 1,818 (1,768+50) | - | 1 (Images) |
| Mark Christopher et al. [7] | 2020/4/28 | ResNet^c^ | - | - | 79.0-97.0 | - | 6,721 (5,357+1364) | 19,180 (8,706+1768) | - | 1 (Images) |
| Guangzhou An et al. [8] | 2019/2/18 | Multimodal model (VGG19^b^ as images features extractors) | 96.3 | - | - | - | 208 | 149 | - | OCTA^d^, and fundus |
| ^a^AUROC: area under receiver operating characteristic curve, | | | | | | | | | | |
| ^b^VGG: Visual Geometry Group deep learning network | | | | | | | | | | |
| ^c^Resnet: residue network | | | | | | | | | | |
| ^d^OCTA: optical coherence tomography angiography | | | | | | | | | | |

**References**

1. Li Z, He Y, Keel S, Meng W, Chang RT, He M. Efficacy of a deep learning system for detecting glaucomatous optic neuropathy based on color fundus photographs. Ophthalmology. 2018;125(8):1199-206. PMID: 29506863; doi: 10.1016/j.ophtha.2018.01.023.
2. Christopher M, Belghith A, Bowd C, Proudfoot JA, Goldbaum MH, Weinreb RN, et al. Performance of deep learning architectures and transfer learning for detecting glaucomatous optic neuropathy in fundus photographs. Scientific Reports. 2018;8(1):1-13. PMID: 30420630
3. Al-Aswad LA, Kapoor R, Chu CK, Walters S, Gong D, Garg A, et al. Evaluation of a deep learning system for identifying glaucomatous optic neuropathy based on color fundus photographs. Journal of glaucoma. 2019;28(12):1029-34. PMID: 31233461
4. Hemelings R, Elen B, Barbosa-Breda J, Lemmens S, Meire M, Pourjavan S, et al. Accurate prediction of glaucoma from colour fundus images with a convolutional neural network that relies on active and transfer learning. Acta ophthalmologica. 2020;98(1):e94-e100. PMID: 31344328
5. Phan S, Satoh Si, Yoda Y, Kashiwagi K, Oshika T. Evaluation of deep convolutional neural networks for glaucoma detection. Japanese journal of ophthalmology. 2019;63(3):276-83. PMID: 30798379
6. Shibata N, Tanito M, Mitsuhashi K, Fujino Y, Matsuura M, Murata H, et al. Development of a deep residual learning algorithm to screen for glaucoma from fundus photography. Scientific Reports. 2018;8(1):1-9. PMID: 30279554
7. Christopher M, Nakahara K, Bowd C, Proudfoot JA, Belghith A, Goldbaum MH, et al. Effects of study population, labeling and training on glaucoma detection using deep learning algorithms. Translational vision science & technology. 2020;9(2):27-. PMID: 32818088
8. An G, Omodaka K, Hashimoto K, Tsuda S, Shiga Y, Takada N, et al. Glaucoma Diagnosis with Machine Learning Based on Optical Coherence Tomography and Color Fundus Images. J Healthc Eng. 2019;2019. PMID: 30911364; doi: 10.1155/2019/4061313
