# Supplementary material for: Use of multimodal dataset in AI for detecting glaucoma based on fundus photographs assessed with OCT: focus group study on high prevalence of myopia
Source: BMC Med Imaging. 2022 Nov 24;22:206. doi: 10.1186/s12880-022-00933-z (PMC9700928; doi:10.1186/s12880-022-00933-z)
Supplement: Supplementary file 10 — Additional file 10. Pre-processing method for public datasets testing and training results. Additional File Table 7. The 10-fold cross validation results of Kaggle public datasets. [file 12880_2022_933_MOESM10_ESM.docx]

### Additional File 10: Pre-processing method for public datasets testing and training results


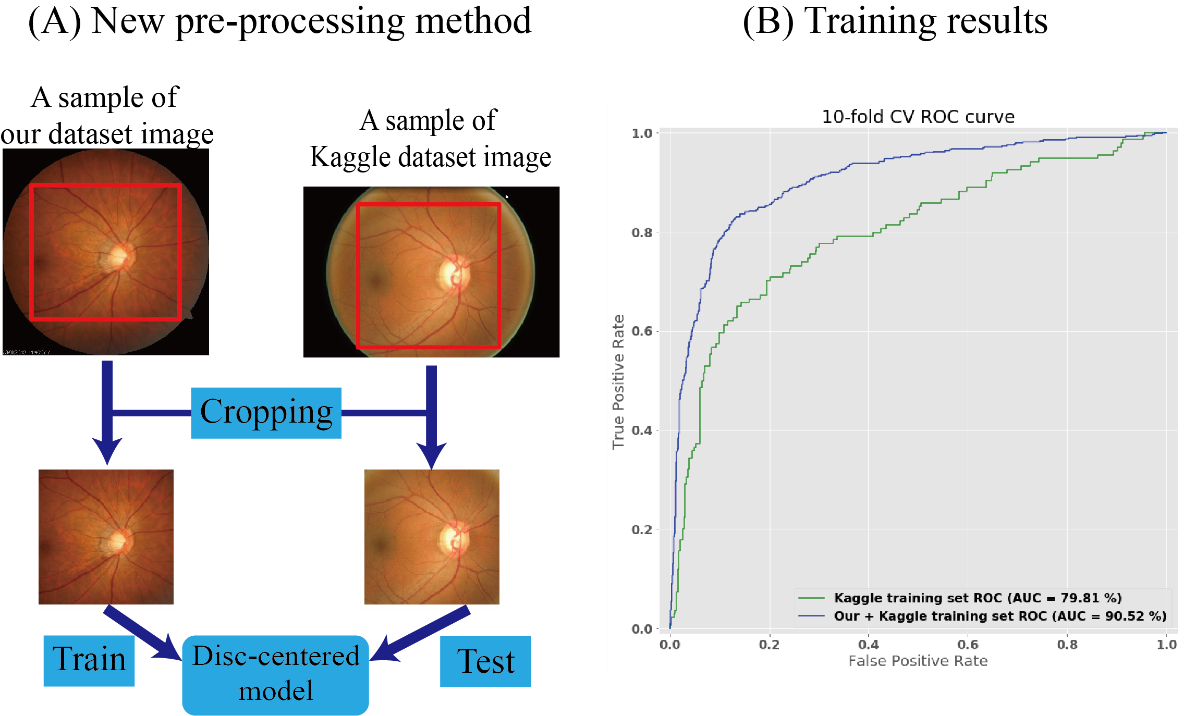


| **Additional File Table 7. The 10-fold cross validation results of Kaggle public datasets** | | |
| --- | --- | --- |
| **Metrics (%)** | **Our dataset + Kaggle (Adapted model)** | **Kaggle only model** |
| **AUROC**^a^**, %** | 90.52 | 79.81 |
| **Accuracy, %** | 84.01 | 73.65 |
| **Precision, %** | 74.61 | 49.25 |
| **Recall, %** | 83.95 | 73.88 |
| ^a^AUROC: area under receiver operating characteristic curve, | | |
