# Supplementary material for: Use of multimodal dataset in AI for detecting glaucoma based on fundus photographs assessed with OCT: focus group study on high prevalence of myopia
Source: BMC Med Imaging. 2022 Nov 24;22:206. doi: 10.1186/s12880-022-00933-z (PMC9700928; doi:10.1186/s12880-022-00933-z)
Supplement: Supplementary file 11 — Additional file 11. Publics dataset testing results and error analysis. Additional File Table 8. The test results of different Kaggle datasets with different models. [file 12880_2022_933_MOESM11_ESM.docx]

### Additional File 11: Publics dataset testing results and error analysis


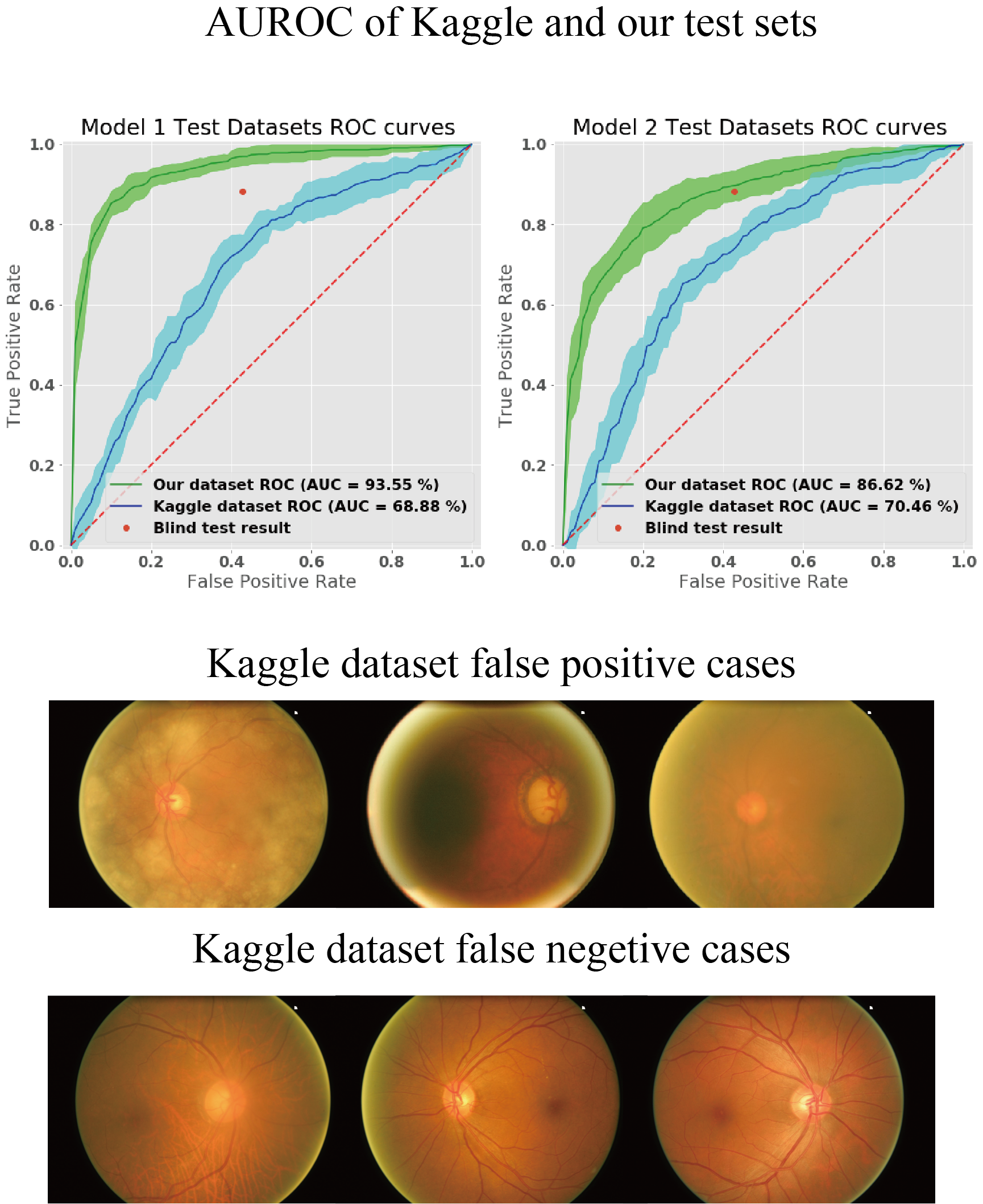


| **Additional File Table 8. The test results of different Kaggle datasets with different models** | | | | | | |
| --- | --- | --- | --- | --- | --- | --- |
| **Metrics (%)** | **Adapted model (model 1) prediction** | | | **Kaggle only model (model 2) prediction** | | **Blind test** |
|  | **our test set** | **Kaggle test set** | **Kaggle dataset 2 test set** | **our test set** | **Kaggle test set** |  |
| **AUROC**^a^**, %** | 93.65 | 68.90 | 83.01 | 86.61 | 70.47 | - |
| **Accuracy, %** | 87.22 | 65.23 | 76.86 | 79.21 | 67.77 | 65.38 |
| **Precision, %** | 88.27 | 40.00 | 69.64 | 80.70 | 42.79 | 42.25 |
| **Recall, %** | 87.20 | 65.00 | 76.91 | 79.24 | 67.35 | 88.24 |
| ^a^AUROC: area under receiver operating characteristic curve, | | | | | | |
|  | | | | | | |
