# Supplementary material for: Use of multimodal dataset in AI for detecting glaucoma based on fundus photographs assessed with OCT: focus group study on high prevalence of myopia
Source: BMC Med Imaging. 2022 Nov 24;22:206. doi: 10.1186/s12880-022-00933-z (PMC9700928; doi:10.1186/s12880-022-00933-z)
Supplement: Supplementary file 12 — Additional file 12. Glaucoma feature labeling tools and guidelines. [file 12880_2022_933_MOESM12_ESM.docx]

### **Additional File 12: Glaucoma feature labeling tools and guidelines**


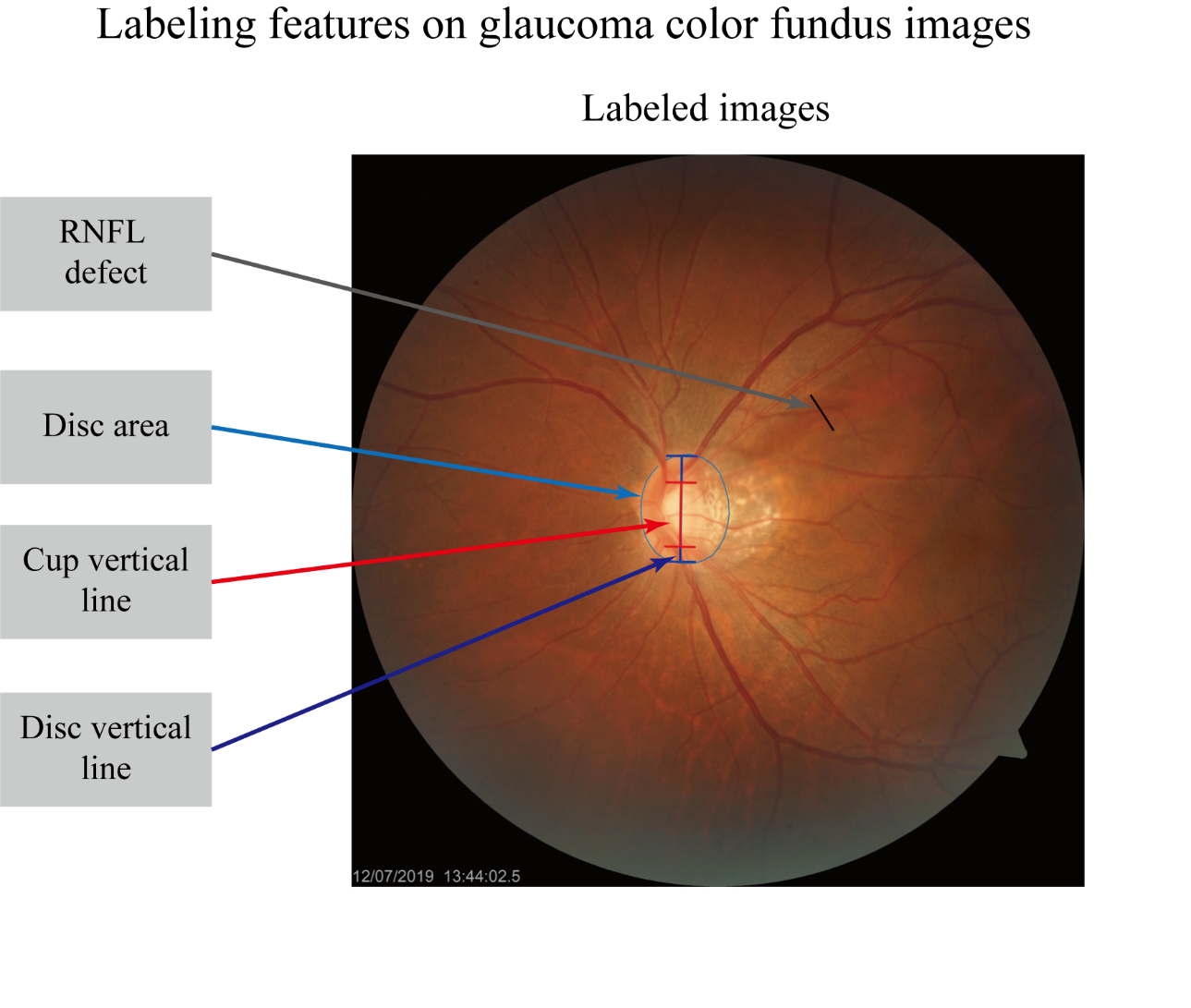
**When training the models, the important glaucoma features on the color fundus images were labeled. The LabelMe tool developed by the Massachusetts Institute of Technology ^1^ was used to label the disc area, disc vertical diameter, cup vertical diameter, and nerve fiber defects on the collected color fundus images. Four well-trained researchers were involved in labeling, and each labeled image was discussed and revised with a senior glaucoma specialist with more than ten years of experience. The labeled data on web pages will be published for academic purposes.** The revised labeled data will be released in JSON format with color fundus images and patients' demographic data for further research.

1. Russell BC, Torralba A, Murphy KP, Freeman WT. LabelMe: a database and web-based tool for image annotation. International journal of computer vision. 2008;77(1-3):157-173.
